# Enhanced efficiency in isolation and expansion of hAMSCs via dual enzyme digestion and micro-carrier

**DOI:** 10.1186/s13578-019-0367-y

**Published:** 2020-01-06

**Authors:** Bi Foua Claude Alain Gohi, Xue-Ying Liu, Hong-Yan Zeng, Sheng Xu, Kouassi Marius Honore Ake, Xiao-Ju Cao, Kai-Min Zou, Sheila Namulondo

**Affiliations:** 1 Biology and Chemical Engineering School, Panzhihua University, Panzhihua, 617000 Sichuan People’s Republic of China; 2 Biotechnology Institute, College of Chemical Engineering, Xiangtan University, Xiangtan, 411105 Hunan People’s Republic of China; 3 Economical Forest Cultivation and Utilization of 2011 Collaborative Innovation Center in Hunan Province, Hunan Key Laboratory of Green, Zhuzhou, China; 4 Packaging and Application of Biological Nanotechnology, Hunan University of Technology, Zhuzhou, 412007 Hunan China; 5 Faculty of Business Administration, Laval University, Pavillon Palasis-Prince, 2325 Rue de la Terrasse, G1V 0A6 Quebec City, Canada; 6 Institute of Comparative Literature and World Literature, College of Literature and Journalism, Xiangtan University, Xiangtan, 411105 Hunan People’s Republic of China

**Keywords:** hAMSCs, Isolation, Dual enzyme digestion, Expansion, Chitosan-based microspheres, Enhanced efficiency

## Abstract

A two-stage method of obtaining viable human amniotic stem cells (hAMSCs) in large-scale is described. First, human amniotic stem cells are isolated via dual enzyme (collagenase II and DNAase I) digestion. Next, relying on a culture of the cells from porous chitosan-based microspheres in vitro, high purity hAMSCs are obtained in large-scale. Dual enzymatic (collagenase II and DNase I) digestion provides a primary cell culture and first subculture with a lower contamination rate, higher purity and a larger number of isolated cells. The obtained hAMSCs were seeded onto chitosan microspheres (CM), gelatin–chitosan microspheres (GCM) and collagen–chitosan microspheres (CCM) to produce large numbers of hAMSCs for clinical trials. Growth activity measurement and differentiation essays of hAMSCs were realized. Within 2 weeks of culturing, GCMs achieved over 1.28 ± 0.06 × 10^7^ hAMSCs whereas CCMs and CMs achieved 7.86 ± 0.11 × 10^6^ and 1.98 ± 0.86 × 10^6^ respectively within this time. In conclusion, hAMSCs showed excellent attachment and viability on GCM-chitosan microspheres, matching the hAMSCs’ normal culture medium. Therefore, dual enzyme (collagenase II and DNAase I) digestion may be a more useful isolation process and culture of hAMSCs on porous GCM in vitro as an ideal environment for the large-scale expansion of highly functional hAMSCs for eventual use in stem cell-based therapy.

## Introduction

Given the unique properties of mesenchymal stem cells’ multipotencity, its capability to differentiate not only into mesenchymal lineage cells but also into a variety of end-stage phenotypes, has raised a promising cellular therapy tool for clinical application [1]. Amniotic fluid (AF) contains multiple cell types derived from the developing fetus [2, 3]. Recent studies show that human amniotic mesenchymal stem cells (hAMSCs) have a similar phenotype to bone marrow-derived mesenchymal stem cells (BMSCs) [4, 5]. Human amniotic stem cells have drawn increased interest because not only are they undifferentiated cells with the ability to differentiate into one or more types of cells as the primary types of the 3 lineages of mesoderm, ectoderm and endoderm [6], but they are also capable of self-renewal [7]. These cells, which are harvested and isolated from human amniotic fluid, can be induced to differentiate under defined culture conditions into osteoblasts, chondrocytes, adipocytes, myocytes, and neuronal cells [8], which is relevant for both scientific and therapeutic purposes. However, the conventional cell-isolation procedure and cell-culture approaches which investigate cellular characteristics on two-dimensional (2D) substrates resulting in anomalous cellular behavior, morphology and physiology [9] should be improved. A major limitation in this evolving discipline is the hardship and the lack of standardization techniques to isolate hAMSCs. Isolation of stem cells is a delicate multi-step process for which protocols must also undergo adjustments according to the source or species of stem cells. Few methods have been described to isolate hAMSCs population; however, none has been proven most effective, mainly due to their effects on proliferation and differentiation capability of the isolated cells. It is hypothesized that our newly established dual enzyme isolation method may provide a better alternative as compared to the standard isolation method. The 3D culture models have proven to be more realistic for translating the study findings for in vivo applications [10] (see Figure S1 on Additional file 1). Human amniotic stem cells may be multipotent but our finite understanding of their behavior in three-dimensional (3D) tissue constructs restricts their therapeutic application. The isolation, generation, and maintenance of stem cells pose several challenges due to the propensity of stem cells to differentiate and for variations such as chromosomal and epigenetic changes to occur in these cells during culture. Protocols are continuously evolving and vary for different types of stem cells. It is necessary to develop new specific protocols for hAMSCs isolation and expansion without any anomalies. In so doing, an increase in the attractive effect of hAMSCs source for potential applications in regenerative medicine and tissue engineering will be realized. This study is carried out with two objectives: to establish an isolation method securing high quantity, purity and activity of hAMSCs and to investigate 3D hAMSCs development on three types of porous chitosan-based microspheres (chitosan microspheres (CM), gelatin–chitosan microspheres (GCM) and collagen–chitosan microspheres (CCM)). The aim is to ensure successful culture as well as exhibit typical stem cell morphology, specific cell surface, and pluripotency markers characteristics.

## Materials and methods

### Materials

Chitosan was purchased from Sinopharm chemical reagent company (Shanghai, China). EDTA and bovine gelatin were obtained from Gibco manufacturer (Peking, China). Trypsin, fetal bovine serum (FBS), collagenase II from *Clostridium histolyticum* lyophilized powder and 10104159001-DNase I from bovine pancreas were purchased from Roche (Basel, Switzerland). Anti-human FITC was purchased from BioLegend, Inc. (San Diego, USA). Rabbit anti-human CD133, Oct-4 and h-TERT were purchased from MyBioSource (San Diego, USA). Collagen type I from bovine calf skin and Dulbecco’s Modified Eagle’s Medium (DMEM)/F12 medium were purchased from Sigma-Aldrich Co. LLC (Peking, China). All other antibodies were purchased from Becton Dickson Co., Ltd (Shanghai, China). The test for Human Immunodeficiency Virus (HIV), infectious syphilis and other related indicators were performed on all the placentas and they tested negative. The chemical reagents, culture medium and antibiotics used in this study were of cell culture grade.

### Isolation of hAMSCs

Amnion tissues were immediately collected from human term placentas of 37 gestational weeks (N = 30) after elective caesarean section. Placentas were collected immediately following delivery and placed into cold phosphate buffered saline (PBS). Samples (about 3 to 5 ml) were placed in a 10 cm sterile Petri dish, and the residual blood clots and amniotic epithelial cells were curetted with the cell scraper. They were then repeatedly washed in cold PBS until the majority of blood was cleared and the cord and membranes removed. The amnion pieces were treated with 0.25% trypsin for digestion to remove the epithelial cells and further treated by 0.02% EDTA (V:V = 1:1) at 37 °C for 60 min. Then a filtration with a 100 mesh cell strainer then followed by digestion of 1.0 g/L collagenase II and 0.1 g/L DNAaseI (V:V = 1:1) at 37 °C and were operated for 60 min. The released cells were filtered with a 300-mesh cell strainer and rinsed with PBS. Centrifugation at 1000 rpm ensued for 5 min. The obtained cells were re-suspended to prepare single cell suspension of 10^6^ cells/ml by taking a clean hemocytometer slide and fixing the coverslip in place. The surface of the slide was cleaned with 70% ethanol and stained with 0.4% trypan blue in PBS. All the steps were carried out under sterile conditions. Initial counts of freshly isolated cells or harvest from amniotic tissue were normalized from equally sized pieces of amniotic membrane.

### Expansion of mesenchymal stem cells

The collected cells were seeded at a density of 5 × 10^6^ cells in 20 ml of media. The medium constituted DMEM supplemented with 100 U/ml penicillin, 0.1 mg/ml streptomycin (Gibco), 3.7 mg/ml sodium bicarbonate, 10 ng/ml epidermal growth factor (EGF) (Peprotech, Princeton, NJ) and 10% foetal bovine serum (FBS) (Gibco). The primary Culture of hAMSCs was based on methods as previously described by Savickiene et al. [8]. Cells were subcultured into higher passages at approximately 80% confluence with 0.25% trypsin and 0.02% EDTA for 1–2 min. The medium of the subculture process was changed every 2–3 days, and the growth of hAMSCs was observed at regular intervals in order to evaluate and observe the biological behavior of adherent cells in vitro. hAMSCs were seeded at a density of 1 × 10^4^ cells/well onto a 96-well plate and cultured for 24 h. (n = 30 wells for each isolation method) then cells counted from expansion of one plate after each passage.

### Proliferation/metabolic cell viability-MTT assay

At passage 5.10^6^ hAMSCs isolated from each kind of both isolation techniques were seeded on plastic Petri dishes for cell metabolic activity and proliferation assays. Cell proliferation was assessed by MTT (3-(4, 5-dimethyl-thiazol-2yl)-2,5-diphenyl tetrazolium bromide) assay after 48 and 72 h of culture. The assay was carried out in 96-well plates; each well containing the cells to be tested with cultured medium or rinsing solution removed. 10 μl of 5 mg/ml MTT solution was added to each well and incubated for 4 h at 37 °C and then 100 μl of DMSO was added and mixed thoroughly to dissolve the dark blue crystals. After a few minutes, the plates were read on a microplate reader (Appliskan, Thermo Scientific, Finland) at a wavelength of 570 nm [11, 12]. These 100 μl Cell suspensions containing hAMSCs numbers ranging from 20 to 50,000 isolated from dual enzyme digestion and collagenase I digestion, then tested using MTT with 3 h incubation. MTT assay was terminated with 150 μl of dimethylsulfoxide (per well, the cells were lysed for 15 min, and the plates were gently shaken for 5 min). The data were normalized to positive control that represented 100% cell viability. To characterize the cellular phenotype, the expression profile of lineage-specific markers was analyzed by flow cytometry. Described briefly by Vulcano et al. [13] and Fei et al. [14], hAMSCs were cultured in control medium for 72 h prior to analysis. hAMSCs were harvested in 0.25% trypsin/EDTA and fixed for 30 min in ice cold 4% formaldehyde. Following fixation, cells were washed in flow cytometry buffer (FCB; 1× PBS, 2% FBS, 0.05% sodium azide). Cell aliquots (1 × 106 cells) were incubated in FCB containing 20 µl monoclonal antibodies and surface markers as it can seen in Additional file 1: Apenndix Table S1A). Passage-3 stem cells were washed with 0.5 ml phosphate-buffered saline (PBS) and harvested with 0.25% trypsin/EDTA for 1–2 min at room temperature. The cells were re-suspended in PBS at a density of 1x 10^6^/ml and centrifuged at 800 rpm for 5 min, this step was repeated thrice. The samples were stained with fluorescein isothiocyanate (FITC)- or phycoerythrin (PE)-conjugated antibodies (see Additional file 1: Table S1A). After washing, cells were analyzed using a BECKMAN flow cytometry (Beckman Coulter, USA). As control, cells were stained with isotype control IgG.

### hAMSCs differentiation in vitro

For cell differentiation, hAMSCs were isolated from both isolation techniques and from each type of chitosan-based microsphere and seeded into a 4-well (3.85 cm^2^) plate (Nunc, Termo Scientifc, Roskilde, Denmark) at a 1 × 10^4^ cells/cm^2^. Each cell population was differentiated in triplicates using undifferentiated cells for controls. Osteogenic differentiation was induced by culturing hAMSCs for up to 14 days in normal culture medium supplemented with 10^−8^ mol/l dexamethasone, 5 μg/ml ascorbic acid 2-phosphate and 10 mmol/l b-glycerophosphate [15]. To observe calcium deposition, cultures were washed once with PBS and stained for 5 min at RT with Alizarin Red S stain (Sigma-Aldrich, Shanghai, China, catalog number: A3882), pH 4.2. Excess stain was removed through several washes with distilled water. To induce adipogenic differentiation, hAMSCs were cultured for up to 2 weeks in a normal culture medium supplemented with 10^−8^ mol/l dexamethasone and 5 g/ml insulin, a slight modification of a previously described protocol [15]. Adipocytes were easily discerned from the undifferentiated cells by phase-contrast microscopy. To further confirm their identity, the cells were fixed with 4% paraformaldehyde in PBS for 1 h at RT, and stained with Oil Red O (Sigma-Aldrich Shanghai, China Trading Co,Ltd) solution (three volumes of 3.75% Oil Red O in isopropanol plus two volumes of distilled water) for 5 min at RT [16]. Chondrogenic differentiation was induced using the micromass culture technique. 10 μl of concentrated hAMSCs suspension was plated in the center of each well and allowed to attach at 37 °C for 2 h. Chondrogenic medium (1% FBS, 0.1 mM dexamethasone, 50 μg/ml ascorbic acid, ITS + 1 [insulin-transferrin-selenium; Sigma], 10 ng/ml TGF-β1 (Sigma-Aldrich Shanghai, China Trading Co,Ltd), and 10 ng/ml in α-MEM) were gently overlaid so as not to detach the cell nodules. The culture was maintained in CM for 3 weeks before analysis [17]. In vitro cells differentiation assay was realized twice. The first time was cells resulting from isolation techniques, the second time was the cells resulting from different types of microsphere culture.

### Preparation of porous microspheres

Chitosan microspheres (CM), gelatin–chitosan microspheres (GCM) and collagen–chitosan microspheres (CCM) were prepared using a combined technique containing emulsification with the chemical cross-linking method proposed by Shanmuganathan et al. [18] and Akamatsu et al. [19] with slight modifications. Some modifications were made to the amounts of the various proteins, oil, speed and time of emulsification. The modifications made up on chitosan microspheres (CM):a solution of 0.3 g of chitosan in 10 ml of 3% acetic acid was stirred then added to a mixed oil phase containing 5 ml of corn oil and 30 ml of porogen solution DMF/PEG200/1,4-butanediol (10 ml of *N*-dimethyl formamide (DMF), 10 ml of Polyethylene glycol 200 (PEG-200), 10 ml of 1,4-butanediol). Span 80 (5% v/v) was used as an emulsifier. The stirring speed was maintained at 500 rpm throughout the process of microsphere preparation for 15 min. Cross-linking of microspheres with glutaraldehyde was carried out in a neutral solution (pH 7) by slow, dropwise addition of 25% glutaraldehyde followed by vigorous stirring for about 3 h at room temperature. For the gelatin/chitosan microspheres (GCM) fabrication, 0.5 g gelatin powder was dissolved in 5 ml triple-distilled water. After agitation for 2 h at room temperature, 2 ml of 5% (w/v) chitosan/0.5 M acetic acid solution was added. The chitosan solution was slowly dropped into a gelatin suspension in the ratio of 2/1 (gelatin: chitosan) and homogenized to obtain a gelatin/chitosan blend. In regards to collagen chitosan microspheres (CCM), a 2% (w/v) collagen/chitosan solution was obtained by simultaneously dissolving collagen and chitosan in a 2% acetic acid solution. The collagen/chitosan ratio was kept constant at 2/1 (w/w). After emulsification for 20 min (1000 rpm) at room temperature for CCM and GCM, a crosslinking procedure of both types of the microspheres was performed by adding a portion of glutaraldehyde. The obtained microspheres were centrifuged, repeatedly washed with distilled water and acetone, and dried in a vacuum. The dried microspheres were stored at 4 °C prior to usage.

### Characterization of chitosan microspheres

The morphological properties of the microspheres were observed using S-3000 N scanning electron microscopy (SEM, Hitachi, Japan). The particle size distribution and other relative parameters of the microspheres were measured using a laser diffraction particle size analyzer (MS-2000, Malvern, Shanghai, china). All the parameters were calculated based on the average results of three measurements. Twenty microspheres of each type were analyzed to obtain the mean (±) of each parameter.

### hAMSCs culturing on porous chitosan-based microspheres

The dry microspheres (0.1 g) require pre-treatment to be used for hAMSCs culture. The microspheres were rehydrated overnight in 40 ml of PBS (pH = 7.4) at room temperature. The suspension of microspheres in PBS was sterilized by autoclaving at 121 °C for 15 min. After autoclaving, the microspheres were stored in a sanitized workstation and dried under atmospheric vacuum from the workstation before use. The dimensions of the microspheres after drying were compared with those before sterilization and the results proved that the microsphere dimensions before sterilization and after drying were the same. The sterilized microspheres were washed once with PBS and twice in DMEM/F12 medium before being transferred into a fresh culture medium containing 10% FBS and stored at 4 °C. The three types of pre-treated microspheres (2 mg/ml) were seeded with hAMSCs at a density of 1 × 10^6^ cells/ml in DMEM/F12 medium supplemented with 10% FBS in 96-well plates (for each type of microspheres and control) under sterile conditions. After the cell seeding procedure, the suspension was continuously agitated at 50 rpm during the culture process. Regular hAMSCs growth was observed using inverted microscope observation. The plates of cells resulting from dual enzyme digestion without incorporation of microspheres were used as a negative control. Cells were counted from expansion of one plate after each passage, 30 of each type of microspheres were used for this experiment. The count of the number during the doubling-time test was carried out taking 1 ml of chitosan-based microspheres which was diluted into 9 ml physiological saline, then broken by a 30 s treatment with a tissumizer. A 1 min treatment was required to break the more resistant chitosan-based beads. The dilutions were then plated on tissue culture plates and the cells counted. This process was repeated at appropriate time intervals for each type of test.

### Statistical analysis

Data was obtained from at least thirty replicate tests and then presented as mean ± standard deviation. Statistical analysis was carried out by means of Student’s t-tests One-way ANOVA using SPSS 11.0. Value of p < 0.05 and p < 0.01 were considered statistically significant (see Additional file 1: Appendix). Initial counts of freshly isolated cells from amniotic tissue were normalized to the amount of starting material before each comparative test. In addition to the amount of starting stem cells that was the same during all of the comparative tests, the cells chosen represented 100% cell viability from isolation by each technique (dual enzyme digestion technique and collagenase I digestion technique).

## Results and discussions

### Results

#### Isolation and primary culture of hAMSCs

**Fig. 1:**
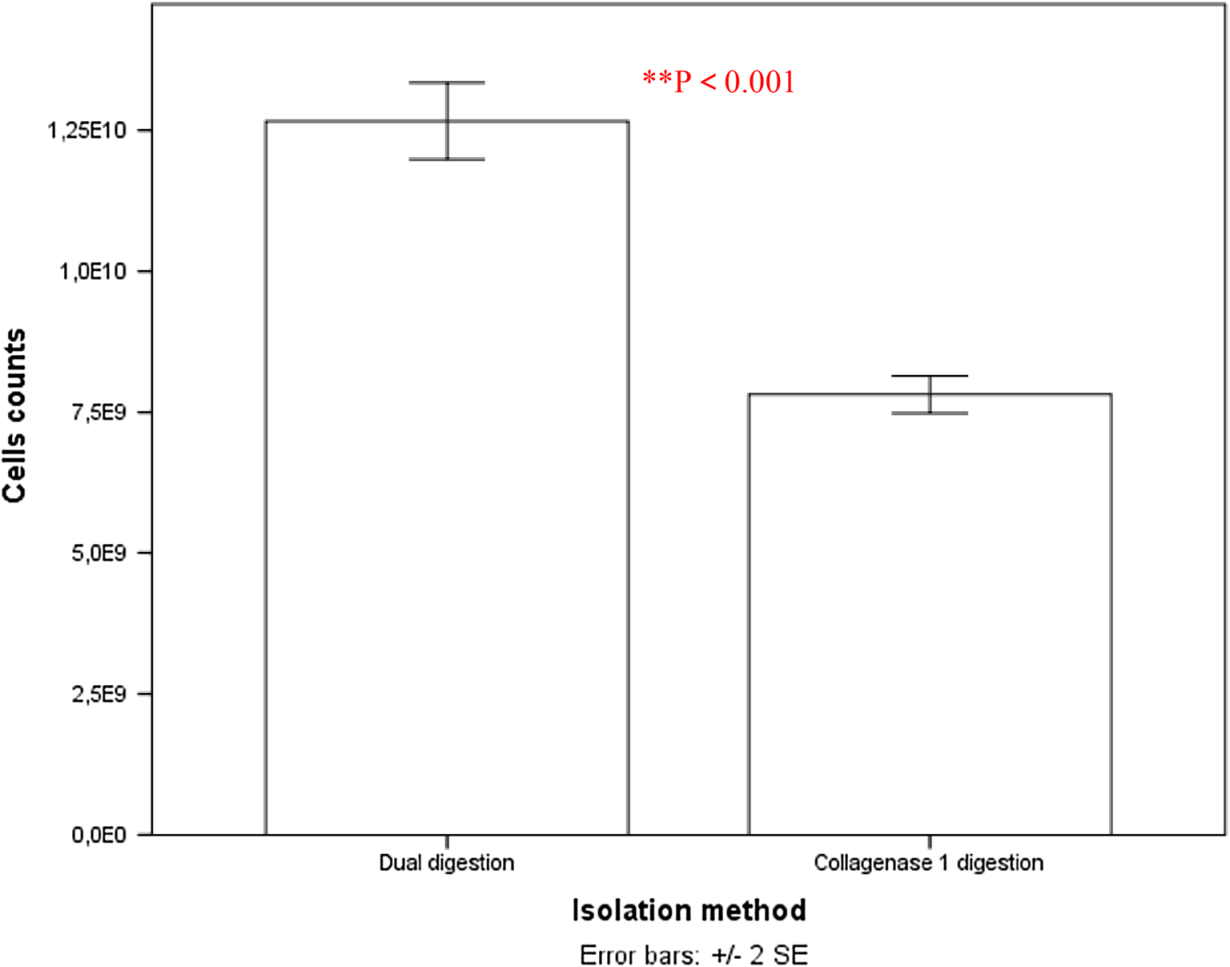
Isolation efficiency of hAMSCs using Dual enzyme digestion and collagenase I digestion processes respectively. Data from cell counts immediately after extraction from one piece of tissue per placenta (N = 30 total placentas) and each cell suspension (n = 30 total suspensions) of each placenta followed by four serial passages of 5 × 10^6^ cells seeded onto tissue culture plates. Live plus dead hAMSCs were counted in a haemocytometer. Mean values ± SE (n = 30) represented by the bars were calculated using SPSS software, statistical significance is indicated (*p < 0.05; **p < 0.01)

We undertook a head-to-head comparison of an existing collagenase I digestion isolation protocol [20] and a dual enzyme digestion process using collagenase II and DNase I. To test for the comparative efficacy of the various enzyme isolation processes, we determined the cell proliferation yield and viability for each method. We investigated the effects of different processes on the isolated cell yields, and following four serial passages in tissue culture flasks, final yields of 12.67 ± 1.9 × 10^9^ and 7.82 ± 0.91 × 10^9^ cells/g were obtained for the dual enzyme digestion and collagenase I digestion processes respectively. There was a significant difference between the yield averages of isolated cells by the dual digestion and collagenase I digestion methods. Dual enzyme (collagenase II and DNAase I) significantly increased the number of isolated cells (p < 0.01, Students t-test; n = 30, hAMSCs were extracted from one piece of tissue per placenta and each cell suspension of each placenta was counted in a haemocytometer), see Fig. 1 and Additional file 1: Appendix Test 1.

**Fig. 2:**
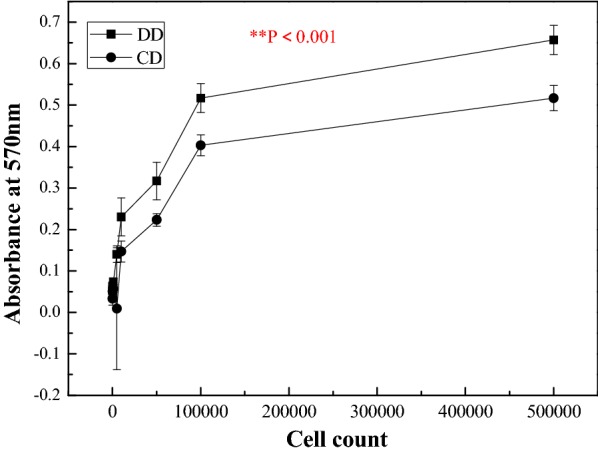
Effects of each isolation technique on viability of hAMSCs by MTT assay, results are expressed as optical density (OD) of healthy cells. Data was derived from cells plated on tissue culture plates and expressed as the mean ± SE of absorbance at 570 nm from at least three independent experiments. Values of *p < 0.05 and **p < 0.01 were considered statistically significant

The number and quality of viable, healthy hAMSCs cells were measured using MTT. The MTT assay showed significantly higher absorbance for cells from dual enzyme digestion versus the control, which is a single collagenase I digestion method after 3 h incubation (p = 0.001) (Fig. 2 and Additional file 1: Appendix Test 2).

**Fig. 3:**
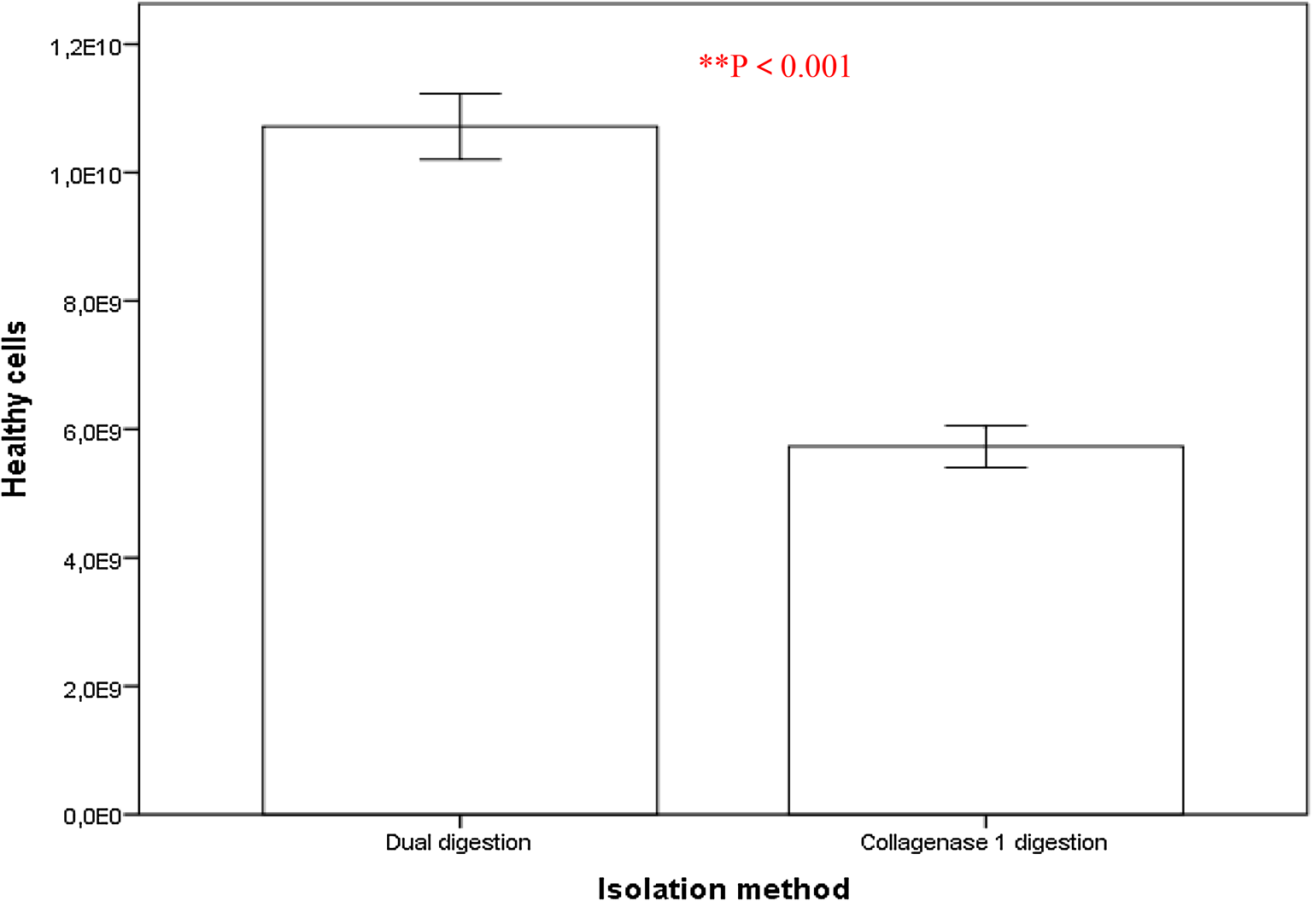
Proliferation of hAMSCs isolated by dual enzyme digestion and collagenase I digestion methods after 2 weeks of 5 × 10^6^ cell culturing on tissue culture plates. Living hAMSCs counted in a haemocytometer. Mean values ± SE (n = 30) represented by the bars were calculated using SPSS software, statistical significance is indicated (*p < 0.05; **p < 0.01)

This is explained by the good dose response of fluorescent/absorbance value to the cells from dual enzymes method. Dual enzyme (collagenase II and DNAase I) digestion method ensured high purity which could imply a low contamination rate of hAMSCs [21, 22]. Concerning the quantitative evaluation of viable cells, a significant amount of viable cells isolated by double digestion was observed compared to cells isolated by a single collagenase I (see Fig. 3, with reference to test 3 of Additional file 1: Appendix (73.27.1% vs. 84.6.3%; P = 0.016 < 0.05, Students t-test; n = 30 Test 3).

**Fig. 4:**
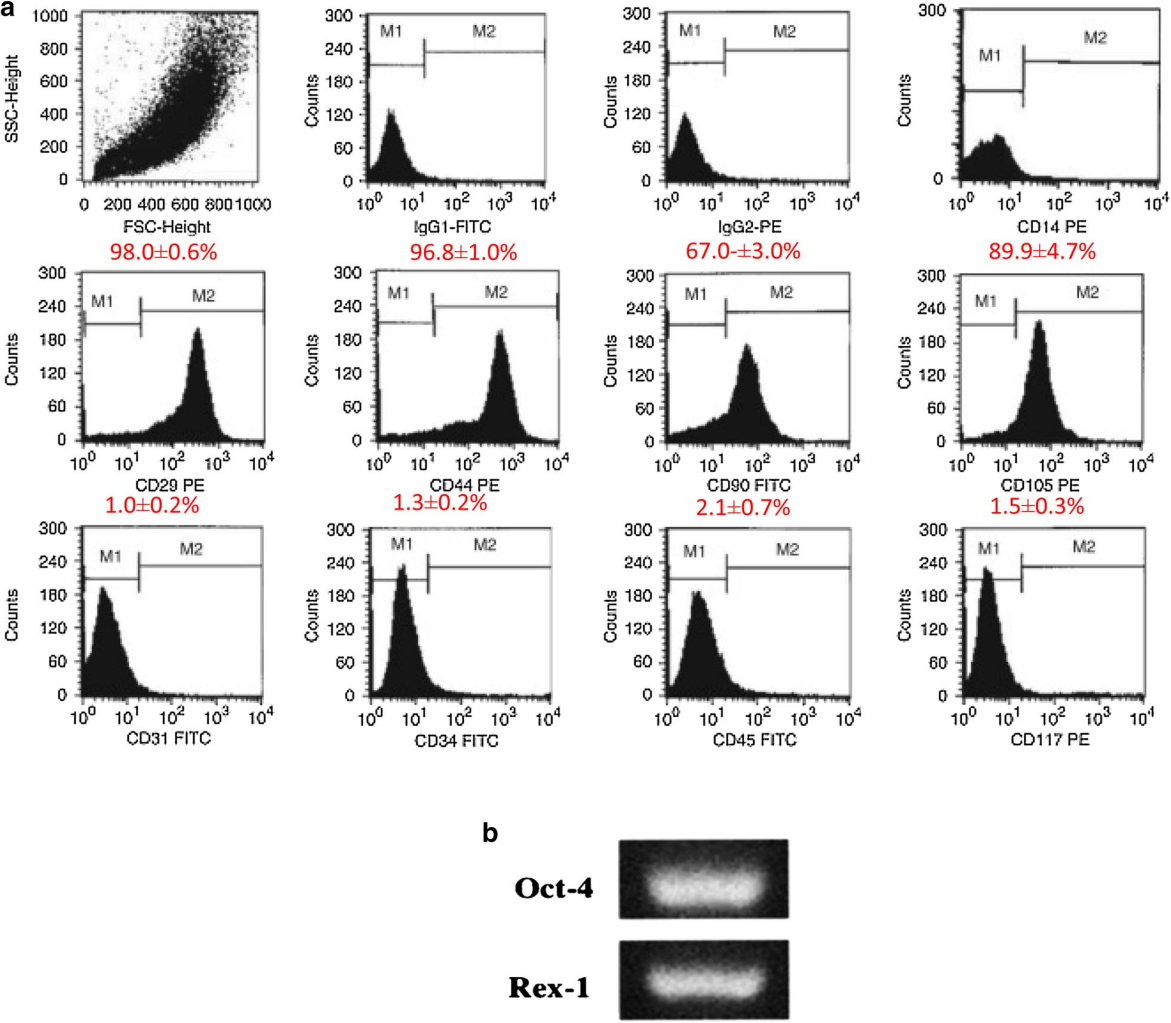
**a** Surface antigen evaluation by flow cytometry. The human amniotic fluid stem cells (hAFSCs) were positive for CD29, CD44, CD90, and CD105; and negative for CD14, CD31, CD34, CD45, and CD117. IgG1-fluorescein isothiocyanate (FITC) and IgG2-phycoerythrin (PE) antibodies were used as isotype controls [30]. **b** Stem cell gene expression. The expression of stem cell genes Octa-4 and Rex-1 [31]

In order to determine whether the dual enzyme digestion process resulted in gross phenotypic changes, we characterized differentiation potential of its hAMSCs ("Differentiation of hAMSCs" secton) and expression of their cell surface markers. Characterization of marker expression of stem cells was realized using the Fluorescence-activated cell sorting (FACS) method [23, 24]). hAMSCs were positive for many surface markers including CD44, CD90 and also positive for transcription factors such Oct-4, Rex1, whereas they were negative for CD34, CD45 and CD117 in agreement with previous reports [25–27]. This confirmed that Dual enzyme (collagenase II and DNAase I) also ensured high activity of hAMSCs. The surface markers of the isolated hAMSC with dual enzyme digestion are all similar to those of the single enzyme method collagenase I digestion technique. The isolated cells expressed representative human amniotic mesenchymal cell markers, especially CD13, CD44, CD73, CD90, CD105, HLA-ABC and Oct-4 but not CD34, CD45 and HLA-DR [8, 28, 29]. The adherent amniotic fluid stem cells were positive for the surface markers that are characteristic of human mesenchymal stem cells such as CD29 showing a M2 gate percentage of about 98.0 ± 0.6%, M2 gate of CD44 which represented 96.8 ± 1.0%, CD90, M2 gate was 67.0 ± 3.0%, and CD105, 89.9 ± 4.7%. A small percentage of the human amniotic mesenchymal stem cells expressed CD45 of 2.1 ± 0.7% for hematopoietic lineage, CD34 1.3 ± 0.2%, of hematopoietic SC), CD117 (1.5 ± 0.3%, c-kit), and CD31 (1 ± 0.2%, endothelial cells). A sample of test results on surface markers is shown in Fig. 4 [30]. A more extensive phenotypic profile of hAMSCs sets is listed in Table 1 and Additional file 1: Table S1A (see Additional file 1: Appendix).

**Table 1:** Brief sample of gene expression by hAMSCs

Gene	Type of protein	Function	Cell type specification
Oct-4	Transcription factors	Create induced pluripotent stem cells	Embryonic stem
Rex1	Transcription factors	Defines region for retinal development	Cone photoreceptor

**Fig. 5:**
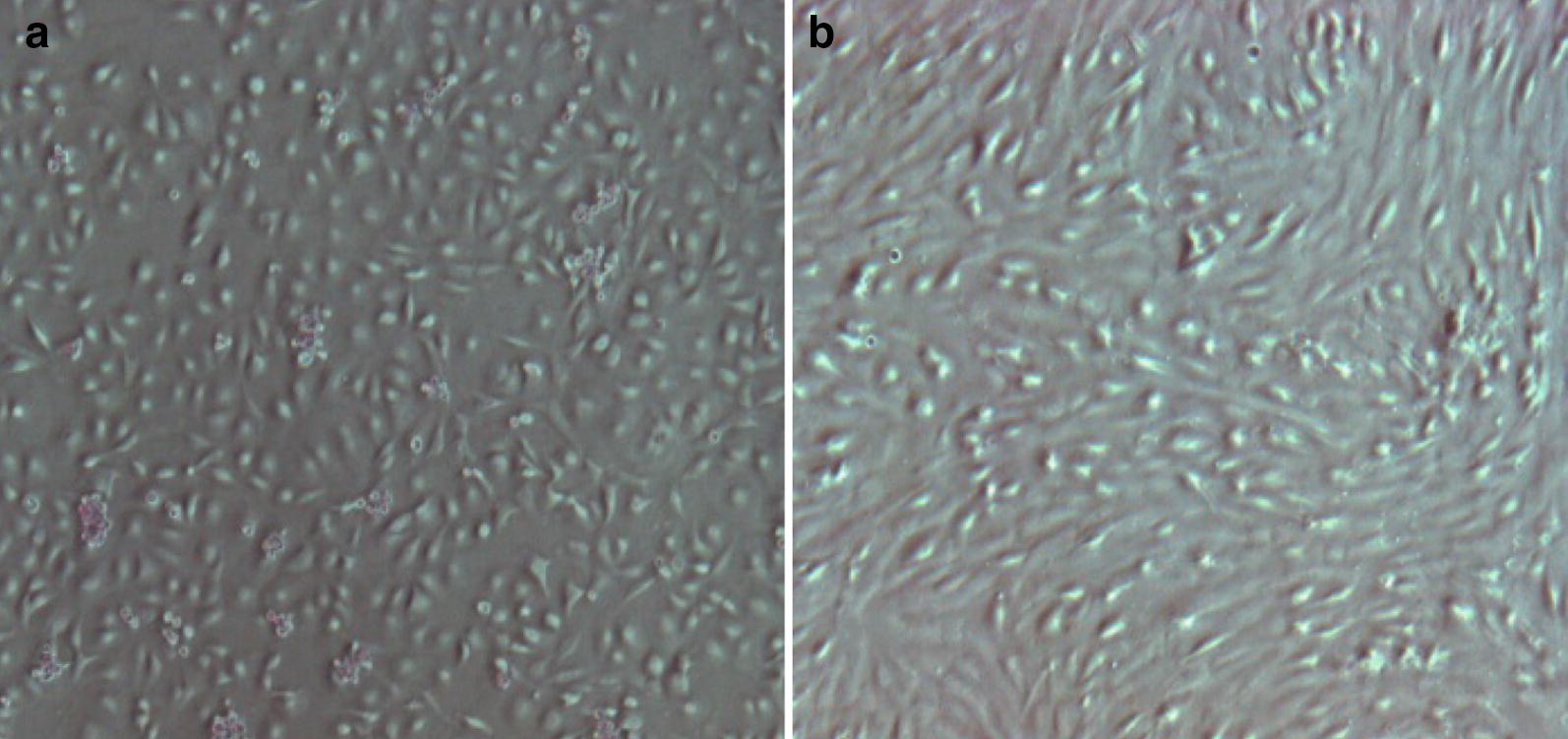
The morphology of primary cultured hAMSCs under inverted microscope (×400), data was derived from 5 × 10^6^ cells plated on tissue culture plates. **a** collagenase I isolation method, **b** dual enzyme digestion isolation method

The cell morphologies are different when the isolated proposed dual enzyme method is compared with the single collagenase I enzyme method. Cells isolated by the collagenase I digestion method were spindle-shaped, polygonal and round-shaped [28, 32] with a round-shaped epithelioid, (which is) the dominant morphology (Fig. 5a). Adherent hAMSCs started appearing 4 to 5 days after isolation by the collagenase I technique. While the resulting cells from the dual enzyme method were found adherent in primary culture after 24 h, the cells resulting from isolation by the double enzyme digestion method were a mixed population with spindle and round-shaped morphology forming colonies, and a dominance of Spindle-shaped cells as can be seen in Fig. 5b [8, 33].

hAMSCs isolated by dual enzyme digestion method presented bipolar morphology, characteristic of immature cells. And seemed acquired a fibroblastic-like morphology. While hAMSCs isolated by collagenase I enzyme digestion method presented spherical cells, which seems to be maternal decidua-derived MSCs. At this stage of isolation, dual enzyme digestion method displayed most cells with a bipolar morphology, characteristic of immature cells, this in contrast to cells isolated by the collagenase I method, which seems to contain a large proportion of already differentiated or adult cells from either a placenta or a fetal. This is confirmed by the differentiation test showing stains for isolated cells by the dual enzyme digestion method which is much more intense than that isolated by the collagenase I digestion method. Although hAMSCs isolated by the collagenase I method normally proliferate as a result of the production of an endogenous factor that promotes growth, the hAMSCs isolated by the double enzyme digestion process proliferate more rapidly, despite the same number of 100% viable cells for both methods at the start of the assay.

#### Expansion kinetic of hAMSCs

**Fig. 6:**
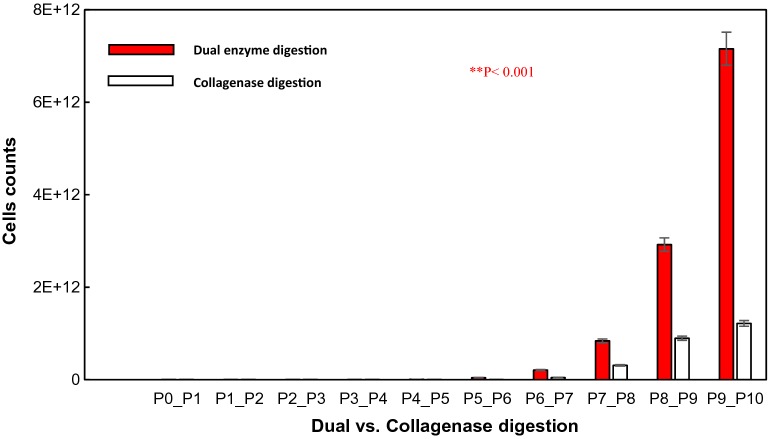
Expansion of hAMSCs isolated from human amniotic membrane by dual enzyme (1) and collagenase I (2) digestion methods. P1, primary cultured hAMSCs, P2-P10, 2–10 passages of subcultured 5 × 10^6^ hAMSCs on tissue culture plates. Results are reported as mean ± SE of the number of live plus dead hAMSCs counted in a haemocytometer (n = 30 assays/group). Statistical analysis was carried out by means of Student’s t-tests, One-way ANOVA using SPSS 11.0. Values of *p < 0.05 and **p < 0.01 were considered statistically significant

MTT (Sigma-Aldrich Trading Co,Ltd (Shanghai, China)) assay was used to measure cell proliferation from different cell passages. Confluent 2nd to 10th passage cells were trypsinized and re-suspended separately [34]. After the first passage, hAMSCs cells showed marked morphological differences. The proliferation capacity of hAMSCs cells was compared at the different passages along the cultivation. hAMSCs expanded staggeringly within a relatively short period of time. Primary cells began to grow adherently at about 30 h, and the logarithm growth phase of cells was stable after a week between the 7th and 10th day. There was a significant difference in cellular proliferation averages between cells isolated by the dual enzyme and collagenase I digestion processes [p < 0.01, (Fig. 6, see Additional file 1: Appendix Test 4)]. In 2 weeks, hAMSCs isolated by the dual enzyme digestion method proliferated faster and on a larger scale than those isolated using the collagenase I method.

**Fig. 7:**
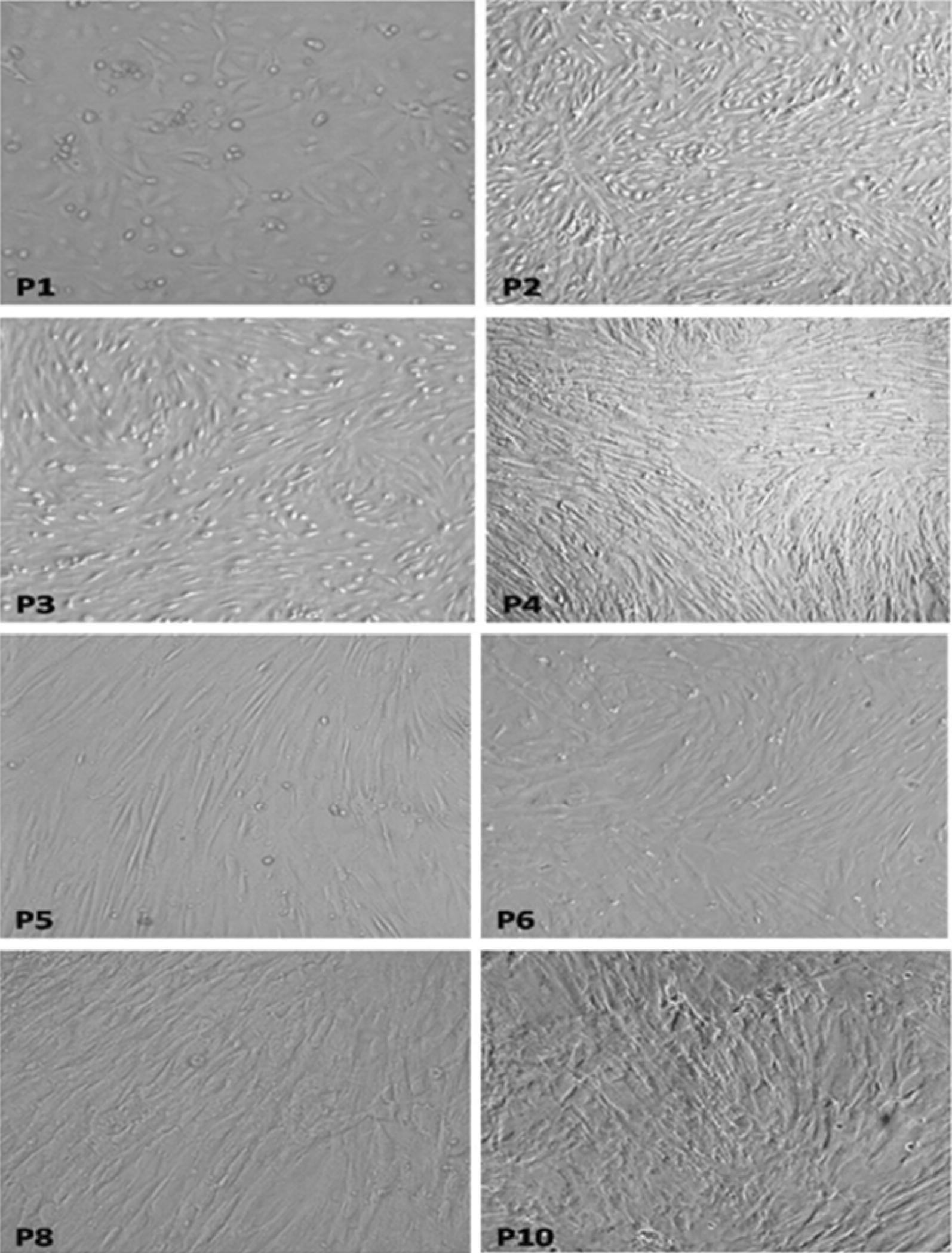
Morphological characteristics of the subculture of hAMSCs isolated using Dual enzyme (×200). P1, primary cultured hAMSCs; P2-P10, 2–10 passages of subcultured 5 × 10^6^ hAMSCs on tissue culture plates

This section of the study focused on cells resulting from isolation by the dual enzyme digestion process because of its high efficiency in both qualitative (high purity of isolated cells Test 2) and quantitative terms (high quantity of viable and healthy isolated cells Test 1 and Test 3). The primary culture contained a gradually growing cell population, which displayed large and flat “stromal” cells with irregular cytoplasmic extensions, and very small nuclei at the edge of the cytoplasm (Fig. 7(P1)). The cells stayed in the detention period after 3 days of passage then turned into the logarithmic phase, and (there is a word missing here) considerably after 7 days. In consequence, cells from the third passage had the greatest proliferation ability (p < 0.05, see Additional file 1: Appendix Test 4), with great growth speed. After 3–8 passages in culture, the population became morphologically heterogeneous with different shape morphologies (Fig. 7(P6)). The proliferation of cells in the tenth generation was slower than in third and fifth generations, see Fig. 6.

#### Differentiation of hAMSCs

**Fig. 8:**
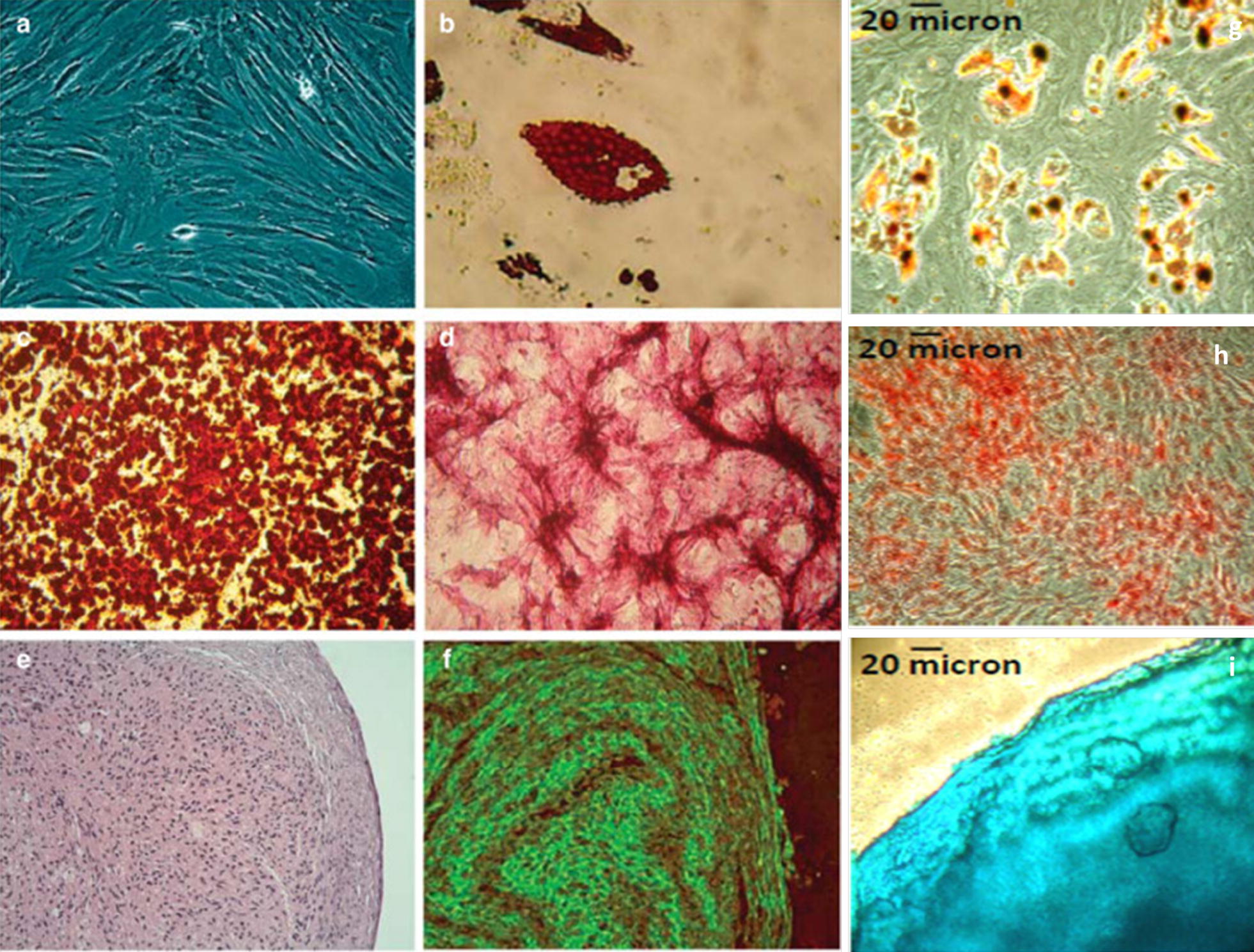
The capacity of human amniotic fluid stem cells (hAFSCs) to differentiate into osteogenic, adipogenic, and chondrogenic lineages. **a** In culture, hAFSCs have fibroblast-like morphology. **b** Adipogenic differentiation was evaluated using Oil Red O staining. Cytoplasmic lipid droplets appear red (×400). **c**, **d** Osteogenic differentiation was confirmed by Alizarin red and alkaline phosphatase staining. Calcium deposits and alkaline phosphatase activity appear red (×100 and ×400, respectively). **e**, **f** Chondrogenic differentiation was confirmed by hematoxylin and eosin and immunofluorescence staining using specific monoclonal antibodies against type II collagen (×200 and ×400, respectively) [30]. Collagenase I isolated cells differentiation figures: **g** Adipogenic differentiation was evaluated using Oil Red O staining. **h** Osteogenic differentiation was confirmed by Alizarin red and alkaline phosphatase staining. **i** Chondrogenic differentiation was confirmed by staining of Alcian blue [35]

To investigate multi potential differentiations, hAMSCs were cultivated in a specific induction as described in "Proliferation/metabolic cell viability‑MTT assay" section (hAMSCs differentiation assays) for to evaluate their ability to differentiate towards adipogenic, osteogenic and chondrogenic lineages. Cells cultured for 2 weeks under adipogenic conditions accumulated lipid vacuoles and exhibited intense staining with Oil Red O. hAMSCs obviously changed after incubation in osteogenic induction medium for 14 days and the cells were capable of osteogenic differentiation as they were positive with Alizarin Red staining. Chondrogenic differentiation was determined after 3 weeks by the appearance of chondrogenic pellets and the production of glycosaminoglycan detected via Alcian Blue staining. The intensity of the Alcian Blue staining was markedly stronger. The three types of differentiation (adipocyte, chondrocyte and osteogenic) was detected after isolation by dual enzyme (collagenase II and DNAase I) digestion as can be seen Fig. 8.

Compared with the hAMSCs isolated by collagenase I method, a precocity in the differentiation potential of about a few hours at the level of the cells isolated by dual enzyme digestion isolation method was noticed. Three types of cell lineages (adipogenic, osteogenic and chondrogenic) were differentiated in addition to a stronger intensity of the makers’ expression of cells isolated by the double enzyme digestion method. The adipogenic differentiation proportion was ranging from 2.87 to 3.2%, osteogenic differentiation level was around 3.7 ± 0.8% and chondrogenic differentiation was of 4.2%. The cells isolated by the collagenase I method showed a more pronounced tendency to differentiate in one of the three types of cell lineage: adipogenic proportion of 2.05%, although they also differentiated into osteogenic (1.15%) and chondrogenic (0.7%) lineages which remains low in intensity.

#### Characteristic analysis of chitosan-based porous microspheres

**Fig. 9:**
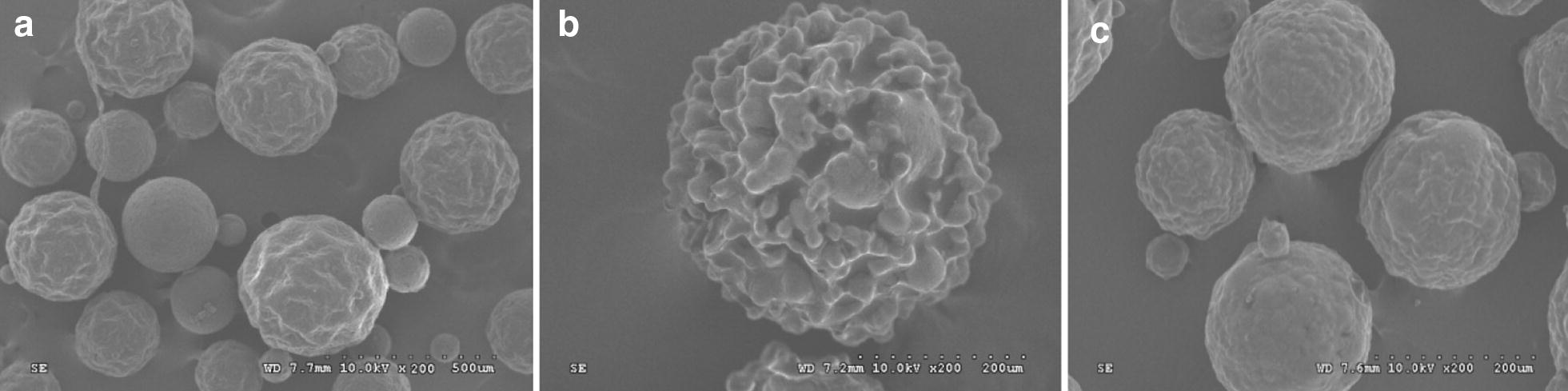
Morphology of porous microspheres by SEM (x 200) in a dry state. **a** CMs; **b** GCMs; **c** CCMs

**Fig. 10:**
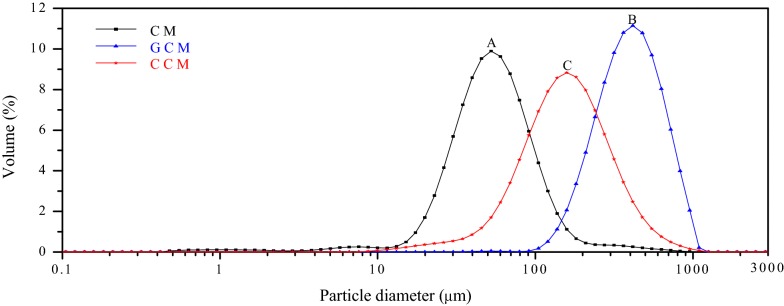
Particle size (μm) distributions of porous microspheres (n = 20 of each type) in dry state. Data is derived from a laser particle analyzer after Sieve analysis method

Polymer microspheres are a popular choice for tissue engineering applications due to the versatility of available materials and tailorable microsphere properties including size, porosity, surface characteristics, permeability, cell adhesivity, and degradation rates [36]. The Chitosan was chosen as a starting material for the synthesis of polymeric microspheres since it displays advantageous biomedical properties. Indeed, chitosan is well known as a biodegradable and biocompatible polymer [37, 38]. The prepared microspheres were a kind of yellow, spherical powder and with good dispersion qualities (Figs. 9, 10).

Many tissue engineering strategies have been developed using microspheres, which are typically defined as spherical or approximately spherical particles with diameters on the micron scale ranging from 1 to 1000 μm [39].

The SEM showed that the microspheres were spherical in shape, well dispersed, with folded surfaces and different sizes depending on the type of microsphere. As shown in Fig. 9a–c, the surfaces of GCMs were much rougher than those CMs and CCMs. This roughness is attributed to the use of glutaraldehyde as a cross-linker [40]. The chitosan microspheres (CMs) were smaller, denser, stronger and more uniform. As for the gelatin chitosan microspheres (GCMs), their surfaces were loose with scattered size distribution and had the largest sizes overall. Collagen chitosan microspheres particle sizes were the most uneven. We concluded that the addition of collagen and gelatin might modify the size distribution, surface area and volume properties of chitosan-based microspheres (Fig. 10).

#### Growth of hAMSCs cultured on porous chitosan microspheres

**Fig. 11:**
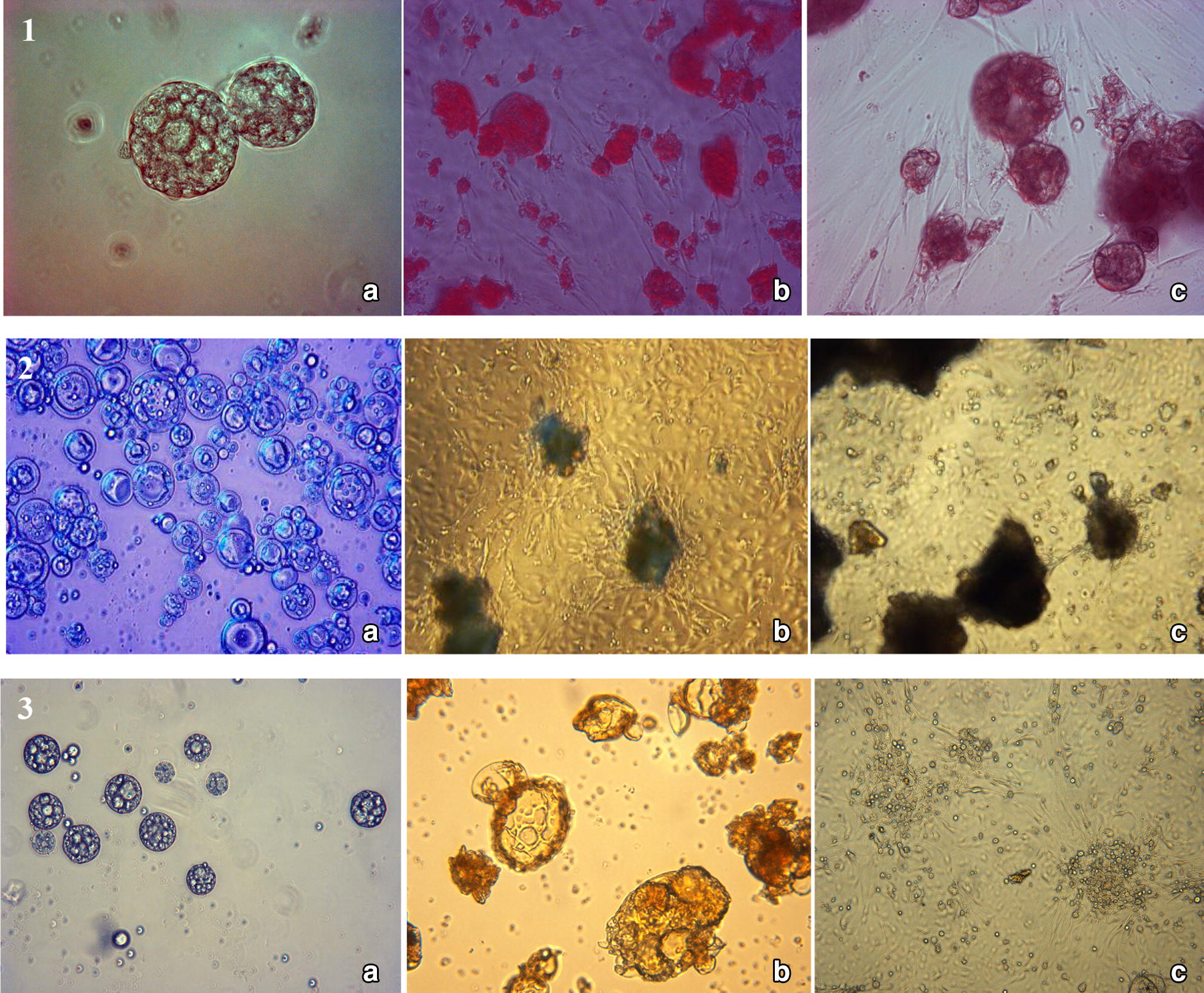
Porous microspheres cultured hAMSCs isolated from human amniotic membrane by Dual enzyme method morphology 1. CMs; 2. GCMs; 3. CCMs. **a** pre-treated microspheres; **b** 3 days of incubations; **c** 7 days of incubation. Cells were seeded at a density of 1 × 10^6^. Live plus dead hAMSCs isolated from the dual enzyme digestion were counted in a haemocytometer. Scale bars **a** = 400 nm; **b**, **c** = 100 nm

In order to facilitate culture of large-scale hAMSCs, we undertook cultures on chitosan-based microspheres [1, 41–43]. The cell expansion profiles for CMs, CCMs and GCMs are shown in Fig. 11. The microspheres were washed with sterile phosphate-buffered saline (40 ml PBS solution of pH = 7.4 at 37 °C) and centrifuged again (1500 rpm for 5 min) to remove the supernatant. Sterile PBS was added to the centrifuge tube to resuspend the microspheres. The tube with the microsphere suspension was stored at 4 °C before the cells were seeded. Three types of pre-treated microspheres (2 mg/ml) were seeded with hAMSCs at a density of 1 × 10^6^ cells/ml in DMEM/F12 medium supplemented with 10% FBS in 12-well plates under sterile conditions. Extracellular matrix (CMs; GCMs and CCMs) comprised of a rich meshwork of proteins and proteoglycans which did not only contain biological cues [44] for cell behavior [45], but also a reservoir for binding growth factors [46]. Histological analysis and live cell imaging revealed that the cell-chitosan constructs within interconnected porous chitosan and showed significant interaction between the cells and the chitosan-based microspheres [44, 47]. The results indicated that the cells did not show normal monolayer growth, but displayed a spherical shape and aggregated growth on CMs as Figure portrayed by Fig. 11.1b. This could be explained by the fact that chitosan has captured, deformed and aggregated hAMSCs to some extent due to its rigid cross-links [48].

**Fig. 12:**
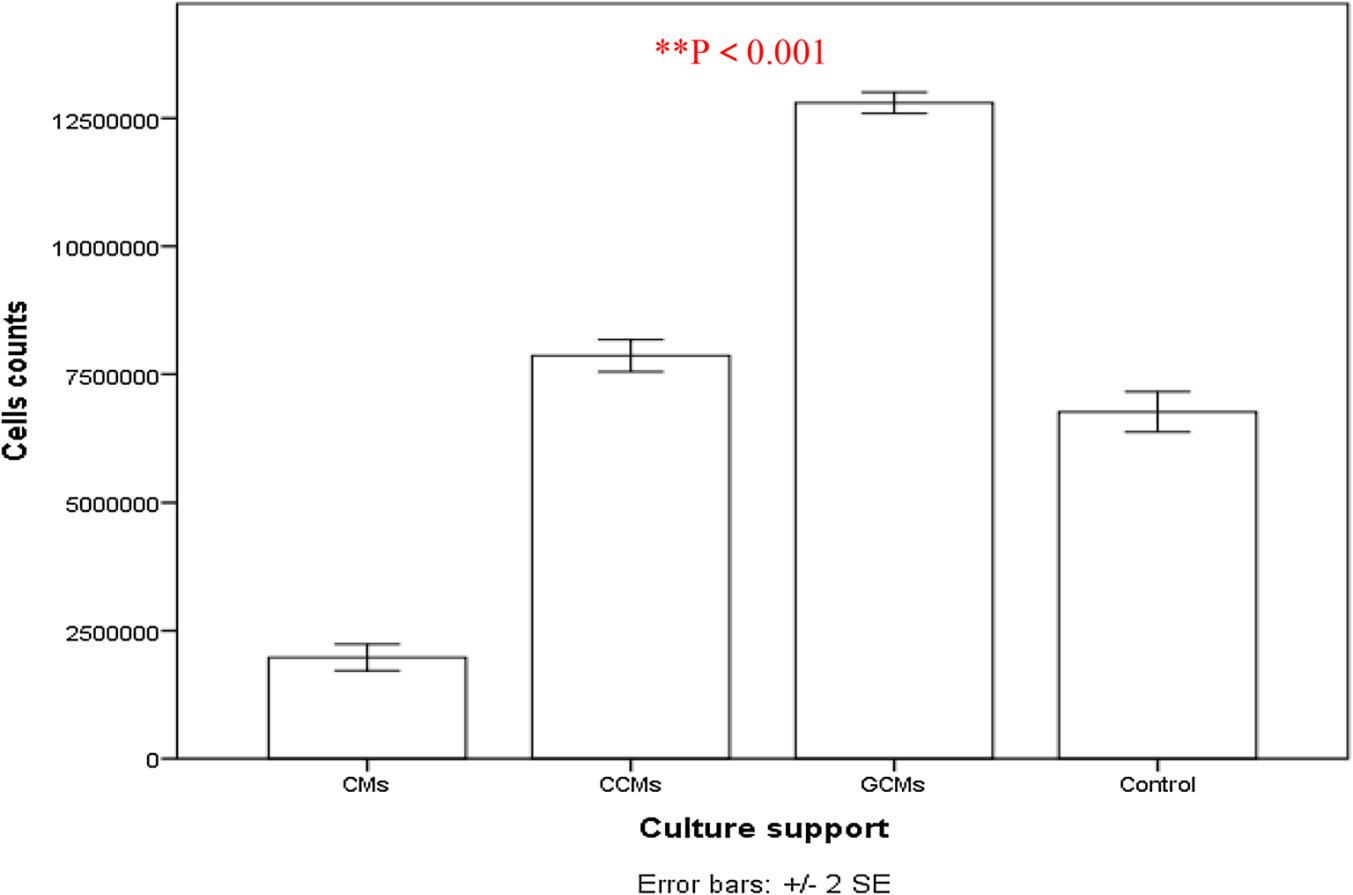
Proliferation of hAMSCs isolated from human amniotic membrane by dual enzyme method during a 7-day culture period (i.e. cell expansion for the 2nd passage of cells) on the different types of microsphere (chitosan-based microspheres (CMs, GCMs and CCMs) and normal plate. Live plus dead hAMSCs isolated from the dual enzyme digestion were counted in a haemocytometer. Mean values ± SE (n = 30 microspheres of each type) represented by the bars were calculated using SPSS software, statistical significance is indicated (*p < 0.05; **p < 0.01)

**Fig. 13:**
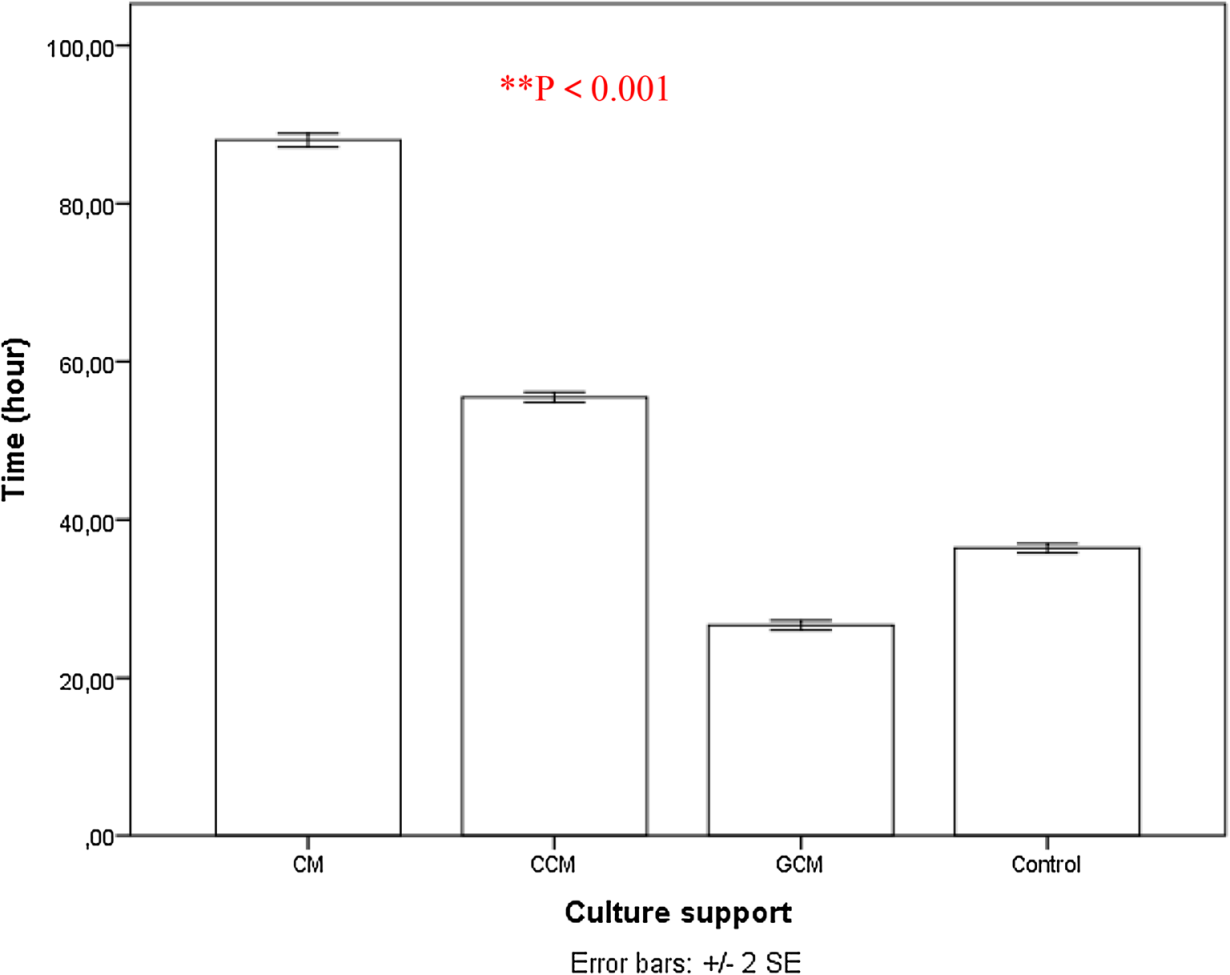
The doubling time in hours (h) for hAMSCs isolated from human amniotic membrane by dual enzyme method at different passages on normal plate and different types of chitosan-based microspheres. Live plus dead hAMSCs isolated from the dual enzyme digestion were counted in a haemocytometer. Mean values ± SE (n = 30 microspheres of each type) represented by the bars were calculated using SPSS software, statistical significance is indicated (*p < 0.05; **p < 0.01)

**Fig. 14:**
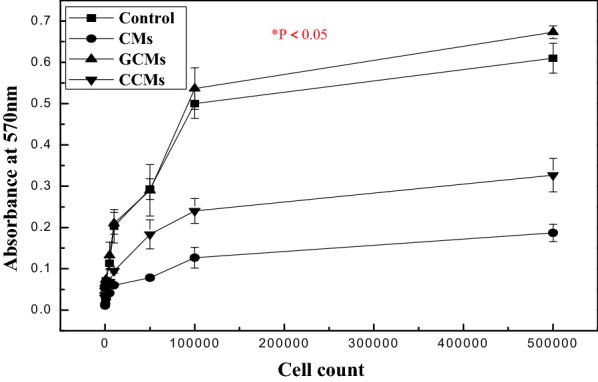
Viability of healthy hAMSCs isolated from human amniotic membrane by dual enzyme method and cultured on normal plate and the different type of microsphere (chitosan-based microspheres (CMs, GCMs and CCMs) measured by MTT. Results are expressed as optical density (OD) of healthy cells. Data was derived from cells plated on tissue culture plates and expressed as the mean ± SE of absorbance at 570 nm from at least three independent experiments. Values of *p < 0.05 and **p < 0.01 were considered statistically significant. It should also be noted that the cells growing on the plastic culture are an integral part of the analysis of Fig. 14

hAMSCs grown on CMs pre-treated microspheres showed insignificant growth in both viable cell count (p > 0.05, Additional file 1: Appendix Tests 5 and 6, Figs. 11 and 12) and cell doubling time (88.02 ± 2.45 h, see Additional file 1: Appendix Test 7 and Fig. 13) as compared with cells grown on the control normal plate (36.4 ± 1.64 h control). The pre-treated GCMs had a porous surface and the average pore size was bigger than the other two microspheres (60 ± 20 μm). The GCMs changed from a round to an irregular rupture-like shape, and showed a degradation trend (Fig. 11.2b). Furthermore, they showed the largest number of hAMSCs around the GCMs. The cells adhered to each other and covered the entire surface of the microspheres (Fig. 11.2c). Moreover, the hAMSCs growth was eugenic and aggregated on CCMs. A large number of cells were attached and covered most of CCM surfaces (see Fig. 11.3b, c). The results indicated that CCMs had a strong adhesive property to adhere to hAMSCs (Fig. 11.3b), and hAMSCs grown on CCMs pre-treated microspheres’s showed significant increase (p < 0.05, Additional file 1: Appendix Tests 5 and 6, Figs. 12 and 14) in doubling time (55.51 ± 1.80 h Fig. 12, Additional file 1: Appendix Test 7) as compared with cells grown on normal plates (36.4 ± 1.64 h Fig. 12).

The doubling times for all types of microspheres of different passages are compared in Fig. 13. The doubling times of GCMs were significantly reduced compared with those of CMs and CCMs in each passage, and also with cells grown on normal plates (36.4 ± 1.64 h Fig. 12) [49]. The doubling time of GCMs was prolonged from the fifth to tenth passages and was further prolonged beyond the tenth passage.

Definitely, during the first passage of expansion, the number of cells GCMs (1.28 ± 0.06 × 10^7^) achieved within 1 week of culture was significantly greater than the number of cells obtained on CCMs (7.86 ± 0.11 × 10^6^) which in turn was greater than the number of cells grown on CMs(1.98 ± 0.86 × 10^6^). The number of cells obtained on GCMs were twice as high as that of the CCMs and ten times greater than that of the CMs (Fig. 13). hAMSCs grown on GCMs pre-treated microspheres showed a significant proliferation rate (p < 0.01, appendices Test 5 and 6) and doubling time (p < 0.01, Additional file 1: Appendix Test 7) in contrast to those on a normal plate (26.67 ± 1.67 h vs. 36.4 ± 1.64 h), CMs and CCMs, see Figs. 12 and 13. It is also worthy to indicate that after 3 days of growing there was clearly a significant number of cells growing on the plastic culture plate after being very rapidly expanded on the different types of microspheres (Fig. 11.1b, 2b, 3b), and cells on the plastic dish were partially (specifically those found at the sites of degradation of microspheres) included in the different analysis. These cells revealed a strong metabolism activity corresponding to a higher proliferation rate as assessed by a second MTT (3-(4,5-dimethyl-thiazol-2yl)-2,5-diphenyl tetrazolium bromide) assay after 72 h of culture on microspheres.

Accordingly, hAMSCs cultured on the gelatin chitosan microspheres (GCMs) showed a higher and more rapid proliferation rate of up to 7 days in comparison with the CMs, GCMs and normal control culture on the Petri dish. After 15 days, its proliferation rate slowly decreased to that of the CCMs and Petri dish, but remained higher than that of the CMs cells (Fig. 14).

The above results indicate that the CMs had a slow degradation rate and poor adhesiveness to hAMSCs, which was not beneficial to cell proliferation leading to a weakness in biological activity. The results also suggested that CCMs and GCMs were favorable to the development of the cells and played a certain role in promoting hAMSCs growth [50]. From the above results it is possible to state that the proliferation rate of hAMSCs grown on the CCMs and GCMs are better compared with the cells grown on Petri dishes. The cells grown on those Petri dishes are better than those grown in CMs, confirming the biocompatibility of fabricated chitosan-based microspheres.

Moreover, after expansion on the chitosan-based microspheres, the hAMSCs underwent investigation to ensure the ability of cell differentiation after culture. The cells resulting from culture on GCMs and CCMs exhibited a differentiation potential qualitatively similar to that routinely observed. Accumulation of lipid droplets was detected in adipogenic cultures. Also, an extensive calcium deposit and glycosaminoglycan expression in pellet cultures indicated strong differentiation potential in osteoblast and chondrocyte. This was not the case for cells grown on CMs microspheres, which in addition to physical deformations only had a deposit of calcium, implying loss of adipogenic and chondrogenic differentiation ability, see Table 3.

The differentiation into adipose cells after day 21 was clearly observable on the culture of GCMs compared to the other two types of microspheres. Culture on CMs did not show adipogenic differentiation while an adepogenic differentiation expression level on CCMs was significantly low compared to the rate on GCMs. As the results, the differentiation expression into adipogenetic cells was 3.87% for GCMs and 1.07% for CCMs, respectively. Measurement of cell proliferation on microspheres CMs and GCMs showed a high differentiation of hAMSCs into bone morphogenic stroma cells compared on CCMs. The differentiation resulted also into osteogenetic cells induction with the proportion ranging from 2.88% for CMs, 3.15% for GCMs and 0.45% CCMs respectively. The culture on microspheres also resulted in their differentiation into elongated appearance shape with the cells exhibiting chondrocyte-like round cells (Fig. 11.2, 3b, c). The level of differentiation into chondrogenic cells CCM and GCM microspheres groups all showed a similar. The proportions of cells positive for chondrogenesis were 3.35% for CCMs, 3.69% for GCMs. There was no statistically significant difference in the percentage of cells expressing chondrogenesis among the microspheres CCM and GCM (p > 0.5). While CM aggregates cultured cells remained undifferentiated-small, rounded cells exhibiting amniotic characteristic cell morphology (Fig. 11.1). This while the 2D culture showed a rate of 2.09, 1.15 ± 0.5% and 1.06% of differentiation in adipogenic, osteogenic and chondrogenic cells, respectively.

Among hAMSCs resulting from culture on the three types of chitosan based microspheres, hAMSCs grown on the surface of gelatin and collagen chitosan-based microspheres showed a significant increase of the metabolic activity compared with the cells cultured in the Petri dish, as assessed by MTT assay for the cells cultured on GCMs, CCMs (Table 3, Fig. 14, Additional file 1: Appendix Test 7). We also found a low metabolism in these cells cultured on CMs and CCMs and compared them with the normal cells culture on Petri dish. The results highlight a better cell-friendly environment for GCMs compared with Petri dishes.

## Discussion

There are already many reports on the techniques of mesenchymal stem cells isolation from the human amniotic fluid [14, 51], as well as several studies on extracellular matrices production, making possible to obtain in sufficient quantities of viable and normally functioning stem cells [52–54]. In our study we compared the commonly used isolation technique with a newer one based on a modified old one. We carried out the intervention of a double enzymatic digestion (collagenase II and DNas I) before a set of extracellular matrix with a chitosan-based microspheres serving as environment for abundant and healthy growth of mesenchymal stem cells.

A modified method was introduced in order to obtain a larger quantity and higher purity of hAMSCs. Additionally, curetting the amniotic membrane-chip with a cell scraper removed 40–60% of the hAECs and unwashed blood clots. Collagenases Type I and II, which are members of Matrix metalloproteinases (MMPs), are secreted cell surface-bound zinc metalloendopeptidases that cleave extracellular matrix (ECM) components [55, 56]. Type I and II of collagenases can mostly be distinguished by their preference, for different substrates. Collagenase I (MMP-1) cleaves a broader range of substrates compared with collagenase II (MMP-8); Collagenase I (MMP-1) cleaves collagens I, II, III, VII, VIII [57], X, and XI [58], gelatin and Clq [59], entactin, tenascin, aggrecan, link protein [60], α2-macroglobulin, Ovostatin [61], IGFBP-3 [57], α1-antichymotrypsin [62] and so on, whereas collagenase II specifically cleaves Collagen I, II, and III, Clqe, aggrecan, α2-Mf, ovostating, α1–1Plh, substrate P [63–67]. Furthermore, it is important to note that collagenase I (MMP-1) prefers type III collagen [68–70] and collagenase II (MMP-8) prefers type I collagen [70–72]. Meanwhile, collagen type I, the most abundant collagen of the human body, is often associated with placenta’s type V collagen. It is interesting to note that all this information shows collagenase II (MMP-8) as the more efficient matrix metalloproteinase in isolating human amniotic stem cells.

The digestion of amniotic membranes was shifted to use collagenase II, which contains greater clostripain activity that is nowadays used for cell isolation [73]. Use of clostripain has shown that it was a very good material for cell isolation [74, 75]. It is generally used for heart, bone, muscle, thyroid, cartilage, and liver cells. Collagens are the major fibrous component of animal extracellular connective tissue, while collagenase is an endopeptidase recognized for digestion of native collagen in the triple helix region [76–78]. Treatment of tissues with crude collagenase II, as well as its mixture of proteolytic activities, provides gentle and selective digestion of the intercellular matrix with little loss of viability or damage to cells [79, 80]. In this case, cells released from the tissue can be easily collected by washing and centrifugation. Collagenase raises the purity of the isolated cells [23]. However, Pountos et al. [23] affirmed that enzymatic treatment could potentially result in alterations to the metabolic profile of the isolated cells. Pardo and Selman [81] also reported that cells were left thermally unstable after MMPs proteolysis of fibrillar collagens were in their triple-helical domain [81]. Knowing the disadvantages of enzymatic isolation of cells by collagenase II, we proceeded to an additional digestion using DNase I. DNase I digestion was an important step in the stabilization of cells against alteration and thermal instability. DNase I has already been used for cell isolation [82, 83]. It particularly increases the purity of the isolated cells [84] by preventing the strong adhesion of cells caused by DNA molecules from the destroyed cells [85]. DNase I sensitivity led to changes in the cells cycle, these changes prevents early replication of the DNAs of the first isolated cells [86]. This situation avoids early contamination of DNA while ensuring cell purity and viability. The culture was not contaminated since the immunocytochemical response was not detected.

It is the combined action of collagenase II and DNase I at the origin that results in both quantitative and qualitative improvement of the human amniotic stems cells’ isolation by dual enzyme digestion. In contrary of Jung and Yoon [87] study, we deduced that the association of collagenase II and DNase I for hAMSCs isolation resulted in isolation of high purity cells.

The method of cell isolation described here using dual enzyme digestion requires a longer digestion time, which is in good agreement with Welgus et al. [69] and Hasty et al. [70] reports. It confirms that collagenase I (k_cat_/K_m_ = 18 M^−1^ s^−1^ 10^−3^) acts faster than collagenase II (k_cat_/K_m_ = 2.5 M^−1^ s^−1^ 10^−3^) on human substrates with a digestion time of 2 h for dual enzyme digestion compared with the 1 h for the methods described elsewhere by Pountos et al. [23], Tuli et al. [24] and that of the Traditional isolation method by Robey [88]. However, the dual enzyme isolation method produces a larger amount of high purity hAMSCs (Figs. 1, 2, 3, 5 and 6, Additional file 1: Appendices Tests 1, 2, 3, 4 and Tables 1 and 2). Isolating cells from soft tissues using collagenase II only results in fragmented cell clusters, low valid cell number, and poor cell integrity in the short run. Collagenase II contains non-protease and protease components, thereby inducing substantial variations in efficacy of the cells isolation [89]. This is why all subsequent studies using collagenase II have associated one or more other enzymes when it comes to cell isolation from soft tissue to overcome the problems of single use of collagenase II as a means of cell isolation [90]. However, single collagenase II can be used when isolating cells from bones or any other hard tissue [91, 92]. Based on the results from mesenchymal markers, hAMSCs isolated from both techniques express more or less different levels of markers associated with pluripotency. Oct-4 embryonic stem cells marker, stage-specific embryonic antigen-4 specific markers of human embryonic cells and Nanog protein, which is a protein responsible for pluripotency [93] were expressed by the isolated cells of both techniques. In fact, hAMSCs isolated by both methods expressed genes characteristic of endodermal, mesodermal, and ectodermal germ layers [94], however, only hAMSCs isolated through dual enzyme digestion method exhibit, under specific culture conditions, which differentiate into hepatogenic, myogenic, and cell neuronal lineages [95]. Expression of sub-population of c-kit (CD117)-positive stem cells isolated from Amniotic membrane of both methods was stronger with stem cells isolated by dual enzyme digestion method. CD117 express the transcription factor Oct-4 and have the potential to differentiate into the three germ layers and form embryoid bodies. They also express surface markers characteristic of MSCs, including CD29, CD44, CD73, CD90, and CD105, and differentiate into adipogenic, osteogenic, myogenic, endothelial, neurogenic, and hepatic lineages. c-kit þ hAFSCs did not induce tumor formation. Through the above, we deducted that amniotic stem cells isolated by dual enzyme digestion method are purer than those isolated by collagenase I digestion method.

**Table 2:** Summary table of geometric parameters of different types of microspheres (n = 20) in a dry state

parameters	Type of microsphere		
	CMs	GCMs	CCMs
Average particle size (μm)	40 ± 6.3	400 ± 47.7	180 ± 14.5
Average pore size (μm)	10 ± 6.02	60 ± 20	30 ± 10.5
Average diameter distribution (μm)	30 ± 7.5	300 ± 52.5	160 ± 21.5
Average surface area (m^2^/g)	0.199 ± 0.15	0.016 ± 0.09	0.049 ± 0.12

ata is derived from laser particle analyzer after Sieve analysis method

Tissue engineering is a rapidly growing and multidisciplinary field showing great promise in creating functional replacements to regenerate impaired tissues [96]. It is implemented by seeding cells onto porous three-dimensional (3D) scaffolds [97–99], followed by in vitro culture, allowing cell adhesion, proliferation, differentiation and neo-tissue genesis by providing an interconnected pore network and an adequate pore surface [100]. Many different types of scaffolds that use varied biomaterials have been developed, specifically: hydrogels, microspheres, porous scaffolds, custom scaffolds, fibrous scaffolds and native tissue scaffolds [101]. Biodegradable microspheres are novel candidate materials and are used to support cell growth. They also have the advantageous ability of maintaining a differentiated cell phenotype and allowing cell expansion due to their high surface area [102]. Approaches related to the culture of hAMSCs using microspheres have exploited a variety of biomaterials such as chitosan [103], collagen [104] gelatin [105, 106] and alginate [107, 108] due to their biocompatibility and high efficiency to integrate with host tissue [109].

hAMSCs as MSCs also adhere to a biomaterial by an indirect mechanism mediated through specific proteins from the serum containing media adsorbed on the material’s surface [110, 111]. Chitosan, collagen and gelatin are derived from natural sources that have been proposed for many regenerative applications on tissue for their key features such as their compatibility with implantation, and their degradability over time [112].

Chitosan is a partially deacetylated derivative from chitin that can produce porous scaffolds with a hydrophilic surface and has cell adhesive/differentiating characteristics [111]. The growth result on CMs indicated that CMs would have captured and make hAMSCs aggregate with a certain degree of cross-linking. CMs and hAMSCs were connected with each other through a newly derived matrix diffused into the microspheres at varying degrees. This led to the cells to be fixed firmly to CMs. The chitosan microspheres had good adhesiveness and biocompatibility [113, 114], but the hydrophilic property was poor. Consequently, the adherent hAMSCs were detached from the microspheres with a dropper at regular intervals. This confirmed Shao et al. [115] and Costa-Pinto et al. [116] studies, which reported that scaffolds produced only with chitosan were more difficult to optimize for tissue applications. Owing to its limited mechanical properties and process ability, CMs are not an appropriate substrate to support the attachment and spreading of cells. This inefficiency of CMs could be due to the deacetylation degree of chitosan (80 to 90% of deacetylation degree) used [117, 118]. After incubating for 7 days, the CMs had almost no degradation trend and were still round spheres; the number of hAMSCs showed no significant increase in Fig. 11. Besides, some small hAMSCs were swollen and deformed at different degrees, probably due to strong adsorption of the hAMSCs caused by the large amounts of positive charge CMs [119, 120]. Cell aggregation becomes too large when cultured with CMs, and the cells inside CMs tend to die due to a lack of oxygen and nutrients [121, 122]. This was not conducive for cell proliferation and could have also lowered the biological activities in Fig. 11.1c. CMs were cytotoxic for hAMSCs and this is in good agreement with Bitencourt et al. [123]. In summation, many studies confirmed that single chitosan microspheres is not a good extracellular matrix for cell growth, so these reports always associate chitosan with other compounds, proteins, carbohydrates, synthetics polymers, and so on [124–126].

One of the advantageous properties of chitosan is its ability to conjugate other compounds to its amino and hydroxyl groups [127]. Due to this property, a large number of chitosan modifications have been successfully achieved for biomedical, clinical and microbiology purposes. Such modifications not only improve its physical and chemical properties, but also increase the applicability of this useful polymer [128, 129]. The cell differentiation test on CMs resulted only in osteogenesis [130], where these cells showed osteogenesis upon staining with alizarin red for calcium deposits (Table 3).

**Table 3:** Control of lineage differentiation of hAMSCs isolated from human amniotic membrane using dual enzyme and cultured on different kinds of chitosan-based microspheres

Microsphere type	Differentiation type		
	Adipogenesis (lipid deposit)	Osteogenesis (calcium deposit)	Chondrogenesis (Alcian blue staining)
CMs	−	++	−
GCMs	+++	++	++
CCMs	+	±	++

−, no detection; ±, low detection; +, detection; ++, strong detection; +++, stronger detection

Collagen is a main structural protein in many tissues and contains a variety of bioactive sites that promote cell attachment [131] and regulate cell differentiation [119]. However, it has low mechanical strength and a rapid degradation rate, which are limiting its commercial use [132]. To overcome these limitations, collagen was combined with chitosan to form CCMs. The pre-treated CCMs structure was dense with an uneven size (Fig. 11.3a). The cells completely covered the surfaces of the CCMs after 3 days (Fig. 11.3b), showing that collagen has low antigenicity and suitable biocompatibility [133, 134]. Also the CCMs had retained their initial forms. From the 3rd to 7th day, we observed vigorous growth of cell aggregation accompanied by advanced degeneration of the microspheres CCMs (Fig. 11.3c). CCMs have cellular affinity and provide the much needed mechanical strength [132]. hAMSCs resulting from the culture with CCMs presented adipogenic, osteogenic and chondrogenic differentiation [132, 135, 136]. The cross-linking method increased the mechanical properties of collagen, however the incorporation of another material formed a composite that has shown the most promise in improving the scaffolds characteristics [137, 138]. Meanwhile, the culture also presented numerous deformed and dead cells. The cells’ death and deformation could be explained by the small size of the CCMs pores (30 ± 10.5 μm), which creates cellular congestion during the cell proliferation process, leading to death of the cells. The size of the pores also plays an important role in the development and viability of stem cells [139, 140]. In contradiction with the observation made by Maa et al. [141]; collagen/chitosan scaffold biodegradation is not a long-term process: 28 days compared with 20 days for this study.

**Fig. 15:**
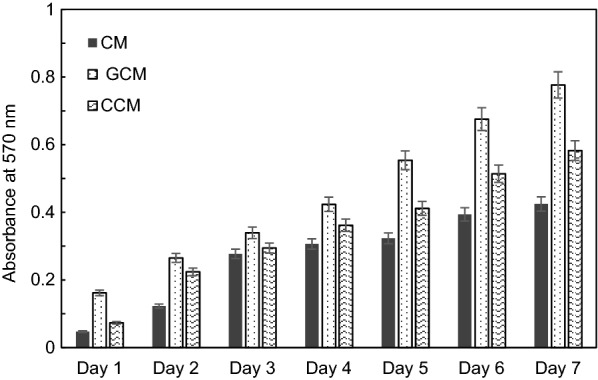
Attachment of hAMSCs on CM, GCM and CCM; asterisk indicates a significant difference in cell attachment. hAMSCs attachment to GCM was significantly higher than both CM and CCM. Data were representative of three independent experiments and all data points were plotted as mean ± SD (n = 10). Comparison between the two means was determined using the Tamhane test of SPSS software, statistical significance is indicated as *p < 0.05; **p < 0.01

The pre-treated GCMs had a porous surface with a larger average pore size than the other two microspheres [60 ± 20 μm (GCMs) > 30 ± 10.5 μm (CCMs) > 10 ± 6.02 μm (CMs)]. This was helpful for the hAMSCs to proliferate effectively into GCMs (Fig. 11.2a) [139, 140, 142]. Gelatin prepared from denaturation of collagen was processed into composites when blended with other materials for promoting cell adhesion, migration, differentiation, and proliferation [124, 143, 144]. GCMs had a stronger adhesive property with hAMSCs than with the other two microspheres (Fig. 15).

Taking advantage of the quantitative analysis of hAMSC growth on different types of microspheres, analysis of the cell adhesion capacity on each type of microsphere was performed. Analysis of the hAMSCs growth attachment capacity on different types of microspheres, CM, GCM and CCM, revealed a significant difference (p = 0.003 < 0.05) in attachment cells to the medium depending on the type of culture medium (Fig. 15, Test 8 in Additional file 1: Appendix). Cell attachment to GCM was sometimes significantly greater than CCM and greater than CM for all time points. In vitro studies, using the GMCs microspheres revealed profound cytocompatibility, increased cell proliferation and enhanced alkaline conditions in GMCs microspheres compared with CMs and CCMs microspheres [105, 106]. Furthermore, observing the resultant cells, protein absorption and mineralization were greater on GCMs scaffolds than on CMs and CCMs microspheres [145, 146]. The growth factor is released as a result of biodegradation of GCMs as in Tabata [147] reported. GCMs were completely covered by hAMSCs, partially degraded on the 3rd day of incubation and utterly degraded after the 7th day. The different phases of Fig. 11 show an increasing cell density from day 1 to day 7 and as can be noticed, GCMs promoted better cell aggregation, which is a crucial step in the initiation of the metabolite process [147, 148]. Cells obtained from culture on GCMs had normal (oval, spherical) form and growth layer [106, 144]. Theywere vigorous and exhibited adipogenic, osteogenic and chondrogenic differentiation [149–152] Table 3. As can be seen from Table 3, the potential for differentiation is influenced by the type of microsphere. It is found that under the same conditions the adipogenesis and Osteogenesis differentiation potential of hAMSCs cultured on GCMs are more successful than on CCMs on one hand, and on the other hand, also under the same conditions the adipogenesis and chondrogenesis differentiation potential of hAMSCs cultured on GCMs are also more successful than those on CMs. After the various tests, we are also noted that stem cell culture on GMCs has several advantages over 2D-control Petri dish (Figs. 12, 13). Indeed, according to the results of the tests, we noticed that the stem cells grew and differentiated more rapidly on GMCs than on 2D-control Petri dish. Starting from the same number of stem cells we obtained more viable cells on GMCs than on 2D-control Petri dish (Fig. 14). Gelatin, the product obtained by collagen denaturation, maintained the biological activity of collagen. Gelatin is conducive, maintains the arrangement of chitosan chains, which made it easier for the microspheres to degrade and be more cohesive to cells. Pore size plays an important role in regulating the fate of mesenchymal stem cells as confirmed by Zhao et al. [97]. The shape of the microsphere influences morphology and metabolism of the cells grown. We suppose that the irregularly shaped cells resulting from culture on the chitosan microsphere were due to unusual metabolic reaction caused by small pore size, surface area and internal interconnection of holes on chitosan microspheres [36]. The shape of the three microspheres changed more or less after 3 days of incubation, and the degree of change was in the following order: GCMs > CCMs > CMs (Fig. 11.1b, 2b, 3b).

The mechanism of microspheres and hAMSCs was explored from the material surface’s structure and composition and could be explained as; a part of the positive charges of chitosan was neutralized by the electrostatic interaction between chitosan–gelatin and chitosan-collagen in the complex microspheres. The absorption capacity of chitosan decreased to a certain extent, changing the strong inherent intramolecular and intermolecular hydrogen bonds and exposing the binding sites of degrading enzyme, thereby enhancing the hydrophilicity of the chitosan fiber. This made it more suitable for cell adhesion, proliferation and enhanced their biological activity [99].

This study has demonstrated that stem cells can be routinely obtained from human amniotic fluid using dual enzyme (Collagenase II and DNAase I) digestion. The obtained cells could be expanded on a large-scale on the porous chitosan-based microspheres. The isolated hAMSCs were viable, pure and in large quantities. They grow easily on GCM microspheres and appear phenotypically and genetically stable. These GCMs have better cell adhesion and proliferation than others [145]. It was shown that mesenchymal stem cells (MSCs) differentiate toward different phenotypes depending on the stiffness of the substrate upon which they are seeded [153], this would explain the difference in the potential differentiation observed on different types of microspheres.

This study ends with both similarity and difference compared to the study of Kim et al. [49]. Similarity regards to “single” chitosan, which is definitely not a suitable biomaterial for cell culture. Rather a contribution than divergence, the gelatin associated with chitosan is more effective than the collagen associated with the chitosan. The nature of the cells in culture, the compositions of the extracellular matrix or both have probably made the difference. The cells’ adhesion to the substrate and cellular behavior depend not only on the chemical composition of the substrate, but also on the surface topography, which defines the organization and physiological activity of cell structures [154, 155].

## Conclusion

In this study, a new attempt has been made on the enhancement efficiency in isolation and expansion of hAMSCs via dual enzyme digestion and microcarrier. We report the isolation of human amniotic stem cells (hAMSCs) by dual enzymatic (collagenase and DNase I) digestion resulting in cells presenting good stability, high viability and expressive embryonic and adult stem cell markers. In conclusion, the new established method, which is the dual enzyme isolation method, is longer as it requires more steps thus more time. However, it is more efficient and produces more viable cells in large quantities than the standard isolation method. Our study also compared the performance of 3D types of chitosan-based microspheres and has demonstrated that hAMSCs can be extensively expanded in vitro on porous chitosan-based microspheres. However, gelatin chitosan microsphere (GCM) was the best choice to culture hAMSCs because it presented the highest degradation ability and the strongest adhesion ability while securing the different qualities of the isolated human amniotic stem cells. GCM maintained a high survival rate and preserves the phenotypic characteristics of hAMSCs. It doubles in 26 h, which is shorter than the other two types of chitosan-based microspheres, and even faster than the traditional monolayer culture system while also being non tumorigenic. The hAMSCs cultured on porous chitosan microspheres offered an accessible method to provide abundant hAMSCs for experimental and clinical use. The mechanism of microspheres and hAMSCs provide the basic theoretical foundation, and highlights the need further investigations to elucidate the mechanism of GCMs during hAMSCs culturing. Consequently, we suggest that GCMs would be a promising extracellular microenvironment for hAMSCs proliferation.

## Supplementary information

**Additional file 1: Figure S1.** Some of the important areas for which 3D cell culture system are excellent models include studies involving drug diccovery, cytotoxicity, genetoxic, cell growth, apoptosis, survival, gene, and protein expression, differentiation and developmental changes, similarity, co-culture in 3D system give a better understanding of the cell interaction [10]. **Table S1.A.** hAMSCs differentiation induction and detection of lineage specific markers after isolation using both methods onto tissue culture polystyrene plates from one piece of tissue per placenta (N = 30 total placentas), and cell suspension (n = 30 total suspensions) per placenta followed by four serial passages of 5 × 10^6^ cells. **Test 1.** Isolation and primary culture of hAMSCs, the yield averages of isolated cells (Fig. 1). **Test 2.** Viability of isolated hAMSCs (Fig. 2). **Test 3.** Proliferation of hAMSCs_Healthy cells (Fig. 3). **Test 4.** Expansion kinetic of hAMSCs, cell proliferation (Fig. 6). **Test 5.** Growth of hAMSCs cultured on porous chitosan microspheres, proliferation of hAMSCs ON CMs, CCMs and GCMs (Fig. 11). **Test 6.** The doubling times for all types of microspheres (Fig. 13). **Test 7.** Viability of healthy hAMSCs isolated from human amniotic membrane (Fig. 14).

## Data Availability

The data and equipment used are presented in the manuscript and also on the additional material.

## Abbreviations

AF: amniotic fluid
AFDSCs: amniotic fluid-derived stromal cells
hAMSCs: human amniotic mesenchymal stem cells
hMSCs: human mesenchymal stem cells
DMEM: Dulbecco’s Modified Eagle’s Medium
FBS: fetal bovine serum
rhbFGF: recombinant human basic fibroblast growth factor
CM: chitosan microspheres
GCM: gelatin–chitosan microspheres
CCM: collagen–chitosan microspheres
SEM: scanning electron microscopy
h: hours
hAECs: human amniotic epithelial cells
MSC: mesenchymal stem cells
DD: dual digestion
CD: collagenase I digestion
