# Supplementary material for: Enhanced efficiency in isolation and expansion of hAMSCs via dual enzyme digestion and micro-carrier
Source: Cell Biosci. 2020 Jan 6;10:2. doi: 10.1186/s13578-019-0367-y (PMC6945441; doi:10.1186/s13578-019-0367-y)
Supplement: Supplementary file 1 — Additional file 1: Figure S1. Some of the important areas for which 3D cell culture system are excellent models include studies involving drug diccovery, cytotoxicity, genetoxic, cell growth, apoptosis, survival, gene, and protein expression, differentiation and developmental changes, similarity, co-culture in 3D system give a better understanding of the cell interaction [10]. Table S1.A. hAMSCs differentiation induction and detection of lineage specific markers after isolation using both methods onto tissue culture polystyrene plates from one piece of tissue per placenta (N = 30 total placentas), and cell suspension (n = 30 total suspensions) per placenta followed by four serial passages of 5 × 10^6^ cells. Test 1. Isolation and primary culture of hAMSCs, the yield averages of isolated cells (Fig. 1). Test 2. Viability of isolated hAMSCs (Fig. 2). Test 3. Proliferation of hAMSCs_Healthy cells (Fig. 3). Test 4. Expansion kinetic of hAMSCs, cell proliferation (Fig. 6). Test 5. Growth of hAMSCs cultured on porous chitosan microspheres, proliferation of hAMSCs ON CMs, CCMs and GCMs (Fig. 11). Test 6. The doubling times for all types of microspheres (Fig. 13). Test 7. Viability of healthy hAMSCs isolated from human amniotic membrane (Fig. 14). [file 13578_2019_367_MOESM1_ESM.docx]

**Appendix**


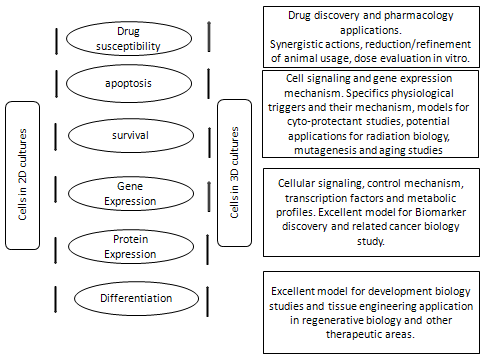


**Figure S1** Some of the important areas for which 3D cell culture system are excellent models include studies involving drug diccovery, cytotoxicity, genetoxic, cell growth, apoptosis, survival, gene, and protein expression, differentiation and developmental changes , similarity, co-culture in 3D system give a better understanding of the cell interaction. [10]

**Table S1.A** hAMSCs differentiation induction and detection of lineage specific markers after isolation using both methods onto tissue culture polystyrene plates from one piece of tissue per placenta (N=30 total placentas), and cell suspension (n=30 total suspensions) per placenta followed by four serial passages of 5×10^6^ cells**.**

| Markers | Anti-body | | | phenotype | |
| --- | --- | --- | --- | --- | --- |
|  | *clone* | *isotype* | *Isotype control* | *positive* | *negative* |
| *CD13* | *WM15* | *Mouse IgG1, κ* | *APC Mouse IgG1, κ Isotype Ctrl* | ***+*** |  |
| *CD14* | *HCD14* | *Mouse IgG1, κ* | *FITC Mouse IgG1, κ Isotype Ctrl (FC)* |  | ***_*** |
| *CD19* | *HIB19* | *Mouse IgG1, κ* | *FITC Mouse IgG1, κ Isotype Ctrl* |  | ***_*** |
| *CD29* | *TS2/16* | *Mouse IgG1, κ* | *APC Mouse IgG1, κ Isotype Ctrl* | ***+*** |  |
| *CD34* | *561* | *Mouse IgG2a, κ* | *APC Mouse IgG2a, κ Isotype Ctrl (FC)* |  | ***_*** |
| *CD40* | *HB14* | *Mouse IgG1, κ* | *FITC Mouse IgG1, κ Isotype Ctrl* |  | ***_*** |
| *CD44* | *BJ18* | *Mouse IgG1, κ* | *FITC Mouse IgG1, κ Isotype Ctrl* | ***+*** |  |
| *CD45* | *HI30* | *Mouse IgG1, κ* | *APC Mouse IgG1, κ Isotype Ctrl* |  | ***_*** |
| *CD49* | *9F10* | *Mouse IgG1, κ* | *APC Mouse IgG1, κ Isotype Ctrl* | ***+*** |  |
| *CD73* | *AD2* | *Mouse IgG1, κ* | *PE Mouse IgG1, κ Isotype Ctrl* | ***+*** |  |
| *CD80* | *2D10* | *Mouse IgG1, κ* | *PE Mouse IgG1, κ Isotype Ctrl* |  | ***_*** |
| *CD86* | *IT2.2* | *Mouse IgG2b, κ* | *APC Mouse IgG2b, κ Isotype Ctrl* |  | ***_*** |
| *CD90* | *5E10* | *Mouse IgG1, κ* | *FITC Mouse IgG1, κ Isotype Ctrl (FC)* | ***+*** |  |
| *CD105* | *43A3* | *Mouse IgG1, κ* | *FITC Mouse IgG1, κ Isotype Ctrl (FC)* | ***+*** |  |
| *CD133* | *MBS767650* | *Polyclonal Rabbit IgG* | *FITC Polyclonal Rabbit IgG* |  | ***_*** |
| *CD144* | *BV9* | *Mouse IgG2a, κ* | *APC Mouse IgG2a, κ Isotype Ctrl (FC)* |  | ***_*** |
| *CD146* | *P1H12* | *Mouse IgG1* | *FITC Mouse IgG1, κ Isotype Ctrl* | ***+*** |  |
| *CD166* | *14-9938* | *Mouse IgG1* | *PE-conjugated mouse monoclonal antibody* | ***+*** |  |
| *BCRP1* | *5D3* | *Mouse IgG2bκ* | *FITC Mouse IgG2b, κ Isotype Ctrl* |  | ***_*** |
| *HLA-DR* | *L243* | *Mouse IgG2a, κ* | *PE Mouse IgG2a, κ Isotype Ctrl* |  | ***_*** |
| *HLA-ABC* | *W6/32* | *Mouse IgG2a, κ* | *Purified Mouse IgG2a, κ Isotype Ctrl* | ***+*** |  |
| *Oct-4* | *MBS695143* | *Rabbit polyclonal IgG* | *FITC Rabbit polyclonal IgG* | ***+*** |  |
| *SSEA-4* | *MC-813-70* | *Mouse Monoclonal IgG3, kappa* | *PE Mouse IgG3, κ Isotype Ctrl* | ***+*** |  |
| *h-TERT* | *Western Blot (WB)* | *Rabbit polyclonal IgG* | *FITC Rabbit polyclonal IgG (Western Blot (WB))* | ***+*** |  |

**- No Detection*

*+ Detection*

**TESTS**

**Test 1**

***Isolation and primary culture of hAMSCs***

- The yield averages of isolated cells (Figure 1)

| **Descriptive Statistics** | | |
| --- | --- | --- |
| Tendance and dispersion | Dual digestion | Collagenase 1 digestion |
| N | 30 | 30 |
| Mean | 12666000000,00 | 7815000000,00 |
| Median | 13500000000,00 | 7900000000,00 |
| Mode | 13500000000 | 8100000000 |
| Std. Deviation | 1871441634,971 | 907576348,076 |
| Variance | 3502293793103450000,000 | 823694827586207000,000 |
| Minimum | 9000000000 | 5950000000 |
| Maximum | 16100000000 | 10000000000 |

| **Independent Samples Test** | | | | | | | | | | |
| --- | --- | --- | --- | --- | --- | --- | --- | --- | --- | --- |
|  | | Levene's Test for Equality of Variances | | t-test for Equality of Means | | | | | | |
|  |  | F | Sig. | t | df | Sig. (2-tailed) | MeanDifference | Std. ErrorDifference | 95% Confidence Interval of the Difference | |
|  |  |  |  |  |  |  |  |  | Lower | Upper |
| Cellscounts | Equal variances assumed | 15,352 | 0 | 12,775 | 58 | 0 | 4851000000 | 379736251 | 4090875306 | 5611124694 |
|  | Equal variances not assumed |  |  | 12,775 | 41,926 | 0 | 4851000000 | 379736251 | 4084621085 | 5617378915 |

**Test 2**

- Viability of isolated hAMSCs (Figure 2)

| **Descriptives statistics** | | | | | | | | |
| --- | --- | --- | --- | --- | --- | --- | --- | --- |
| Absorbance 570nm | | | | | | |  | |
| Cell count | N | Mean | Std. Deviation | Std. Error | 95% Confidence Interval for Mean | | Minimum | Maximum |
|  |  |  |  |  | Lower Bound | Upper Bound |  |  |
| 20 | 6 | 0,043 | 0,01637071 | 0,00668331 | 0,02582 | 0,06018 | 0,02 | 0,065 |
| 100 | 6 | 0,05666667 | 0,01286338 | 0,00525145 | 0,04316737 | 0,07016596 | 0,038 | 0,074 |
| 1 000 | 6 | 0,06566667 | 0,01177568 | 0,0048074 | 0,05330885 | 0,07802449 | 0,05 | 0,082 |
| 5 000 | 6 | 0,11166667 | 0,03399804 | 0,01387964 | 0,07598791 | 0,14734542 | 0,074 | 0,16 |
| 10 000 | 6 | 0,18833333 | 0,05636193 | 0,02300966 | 0,12918512 | 0,24748155 | 0,12 | 0,27 |
| 50 000 | 6 | 0,27 | 0,05932959 | 0,0242212 | 0,20773742 | 0,33226258 | 0,21 | 0,36 |
| 100 000 | 6 | 0,46 | 0,0678233 | 0,02768875 | 0,38882381 | 0,53117619 | 0,38 | 0,55 |
| 500 000 | 6 | 0,58666667 | 0,0821381 | 0,03353274 | 0,50046802 | 0,67286531 | 0,49 | 0,69 |
| Total | 48 | 0,22275 | 0,1974921 | 0,02850553 | 0,16540427 | 0,28009573 | 0,02 | 0,69 |

**Test 3**

- Proliferation of hAMSCs_Healthy cells (Figure 3)

| **Descriptive Statistics** | | |
| --- | --- | --- |
| Healthy cells | Dual Digestion | Coll Digestion |
| N | 30 | 30 |
| Mean | 10717666667 | 5730000000 |
| Median | 10850000000 | 5500000000 |
| Mode | 10900000000 | 4900000000^a^ |
| Std. Deviation | 1402051904 | 882976939,6 |
| Variance | 1,96575E+18 | 7,79648E+17 |
| Minimum | 8100000000 | 4000000000 |
| Maximum | 13600000000 | 7780000000 |
|  |  |  |
| a. Multiple modes exist. The smallest value is shown | | |

| **Independent Samples Test** | | | | | | | | | | |
| --- | --- | --- | --- | --- | --- | --- | --- | --- | --- | --- |
|  | | Levene's Test for Equality of Variances | | t-test for Equality of Means | | | | | | |
|  |  | F | Sig. | t | df | Sig. (2-tailed) | MeanDifference | Std. ErrorDifference | 95% Confidence Interval of the Difference | |
|  |  |  |  |  |  |  |  |  | Lower | Upper |
| Healthycells | Equal variances assumed | 6,144 | 0,016 | 16,49 | 58 | 0 | 4987666667 | 302511587 | 4382123933 | 5593209400 |
|  | Equal variances not assumed |  |  | 16,49 | 48,877 | 0 | 4987666667 | 302511587 | 4379708174 | 5595625160 |

**Test 4**

***Expansion kinetic of hAMSCs***

- Cell proliferation (Figure 6)

|  | **Descriptive Statistics** | |
| --- | --- | --- |
|  | Dual digestion | Collagenase 1 digestion |
| N | 300 | 300 |
| Mean | 1,11736E+12 | 2,47315E+11 |
| Median | 24150000000 | 2504500000 |
| Mode | 6500000 | 4505000000 |
| Std. Deviation | 2,19644E+12 | 4,21683E+11 |
| Variance | 4,82435E+24 | 1,77816E+23 |
| Minimum | 5900000 | 5200000 |
| Maximum | 8,145E+12 | 1,428E+12 |

**Test 5**

***Growth of hAMSCs cultured on porous chitosan microspheres***

- Proliferation of hAMSCs ON CMs, CCMs and GCMs (Figure 11)

| **Descriptive statistics** | | | | | | | | |
| --- | --- | --- | --- | --- | --- | --- | --- | --- |
| Cellular growth | | | | | | |  | |
|  | N | Mean | Std. Deviation | Std. Error | 95% Confidence Interval for Mean | | Minimum | Maximum |
|  |  |  |  |  | Lower Bound | Upper Bound |  |  |
| 1 CMs | 30 | 1976666,67 | 706024,974 | 128901,935 | 1713032,61 | 2240300,72 | 1150000 | 3350000 |
| 2 CCMs | 30 | 7862000 | 855087,816 | 156116,962 | 7542704,96 | 8181295,04 | 6240000 | 9570000 |
| 3 GCMs | 30 | 12800000 | 566294,672 | 103390,789 | 12588542,1 | 13011457,9 | 11500000 | 14000000 |
| 4 Control | 30 | 6764333,33 | 1076471,51 | 196535,91 | 6362372,26 | 7166294,4 | 4650000 | 8600000 |
| Total | 120 | 7350750 | 3947103,43 | 360319,598 | 6637281,21 | 8064218,79 | 1150000 | 14000000 |

| **Test of Homogeneity of Variances** | | | |
| --- | --- | --- | --- |
| Cellular growth | | | |
| LeveneStatistic | df1 | df2 | Sig. |
| 4,352 | 3 | 116 | 0,006 |

| **ANOVA** | | | | | |
| --- | --- | --- | --- | --- | --- |
| Cellular growth | | | | | |
|  | Sum of Squares | df | Mean Square | F | Sig. |
| Between Groups | 1,8E+15 | 3 | 5,918E+14 | 873,792 | 0 |
| Within Groups | 7,9E+13 | 116 | 6,7728E+11 |  |  |
| Total | 1,9E+15 | 119 |  |  |  |

**Test 6**

- The doubling times for all types of microspheres (Figure 13)

| **Descriptives** | | | | | | | | |
| --- | --- | --- | --- | --- | --- | --- | --- | --- |
| Time (hour) | | | | | | |  | |
|  | N | Mean | Std. Deviation | Std. Error | 95% Confidence Interval for Mean | | Minimum | Maximum |
|  |  |  |  |  | Lower Bound | Upper Bound |  |  |
| 1 CMs | 30 | 88,0213 | 2,44675 | 0,44671 | 87,1077 | 88,935 | 79,3 | 92,1 |
| 2 CCMs | 30 | 55,51 | 1,79805 | 0,32828 | 54,8386 | 56,1814 | 49 | 60 |
| 3 GCMs | 30 | 26,6667 | 1,66616 | 0,3042 | 26,0445 | 27,2888 | 24 | 31 |
| 4 Control | 30 | 36,4083 | 1,64412 | 0,30017 | 35,7944 | 37,0223 | 33,5 | 42 |
| Total | 120 | 51,6516 | 23,59564 | 2,15398 | 47,3865 | 55,9167 | 24 | 92,1 |

| **Test of Homogeneity of Variances** | | | |
| --- | --- | --- | --- |
| Time (hour) | | | |
| LeveneStatistic | df1 | df2 | Sig. |
| ,137 | 3 | 116 | ,938 |

| **ANOVA** | | | | | |
| --- | --- | --- | --- | --- | --- |
| Time (hour) | | | | | |
|  | Sum of Squares | df | Mean Square | F | Sig. |
| Between Groups | 65827,465 | 3 | 21942,488 | 5971,232 | ,000 |
| Within Groups | 426,265 | 116 | 3,675 |  |  |
| Total | 66253,730 | 119 |  |  |  |

**Test 7**

- Viability of healthy hAMSCs isolated from human amniotic membrane (Figure 14)

| **Descriptive statistics** | | | | | | | | |
| --- | --- | --- | --- | --- | --- | --- | --- | --- |
| Absorbance 570nm | | | | | | |  | |
|  | N | Mean | Std. Deviation | Std. Error | 95% Confidence Interval for Mean | | Minimum | Maximum |
|  |  |  |  |  | Lower Bound | Upper Bound |  |  |
| 1 Control | 24 | 0,235375 | 0,2024972 | 0,0413346 | 0,149868 | 0,320882 | 0,042 | 0,65 |
| 2 CMs | 24 | 0,068188 | 0,0592733 | 0,0120991 | 0,043159 | 0,093216 | 0,0095 | 0,21 |
| 3 GCMs | 24 | 0,23625 | 0,2174815 | 0,0443932 | 0,144416 | 0,328084 | 0,014 | 0,66 |
| 4 CCMs | 24 | 0,128958 | 0,1059382 | 0,0216246 | 0,084225 | 0,173692 | 0,026 | 0,37 |
| Total | 96 | 0,167193 | 0,1736987 | 0,0177281 | 0,131998 | 0,202387 | 0,0095 | 0,66 |

| **Test of Homogeneity of Variances** | | | |
| --- | --- | --- | --- |
| Absorbance 570nm | | | |
| LeveneStatistic | df1 | df2 | Sig. |
| 16,057 | 3 | 92 | ,000 |

| **ANOVA** | | | | | |
| --- | --- | --- | --- | --- | --- |
| Absorbance 570nm | | | | | |
|  | Sum of Squares | df | Mean Square | F | Sig. |
| Between Groups | ,496 | 3 | ,165 | 6,423 | ,001 |
| Within Groups | 2,370 | 92 | ,026 |  |  |
| Total | 2,866 | 95 |  |  |  |
